# Tough and Rapidly Relaxing Hydrogels Via Programmable Crosslink Kinetics

**DOI:** 10.1002/adma.202523440

**Published:** 2026-04-09

**Authors:** Yuanyuan Wei, Stephen J.K. O'Neill, Jade A. McCune, Oren A. Scherman

**Affiliations:** ^1^ Melville Laboratory for Polymer Synthesis, Yusuf Hamied Department of Chemistry University of Cambridge Cambridge UK

**Keywords:** kinetic programming, programmable time‐dependent mechanics, stress relaxation dynamics, supramolecular hydrogels, toughness

## Abstract

Replicating the synergy of high toughness and rapid stress relaxation found in native tissues remains a central challenge for synthetic hydrogels on account of their intrinsic mechanical–temporal trade‐off. Here we introduce a supramolecular hydrogel platform that leverages kinetic programming to precisely regulate crosslink dynamics through molecular dissociation kinetics. This molecular design allows independent tuning of relaxation dynamics and fracture toughness, decoupling properties that are typically correlated. The resulting hydrogels exhibit stress relaxation ($t_{1/2}$ = 0.1–100 s) two orders of magnitude faster than conventional networks while achieving exceptional fracture energy ($G_{c}=14,500\;J\;m^{-2}$), well above natural rubber. Slowing crosslink dissociation significantly enhances energy dissipation under load, revealing a kinetic principle for toughening viscoelastic networks. This work establishes a molecular blueprint for designing soft materials with programmable, time‐dependent mechanics.

## Graphical abstract

A kinetic programming strategy encodes supramolecular crosslink lifetimes in single polymer networks via stoichiometric control of fast and slow hostguest interactions. This enables predictable stress relaxation on biologically relevant timescales (0.1‐100 s) while maintaining exceptional toughness (up to 14,500 J m^−2^), resolving the long‐standing trade‐off between mechanical strength and dynamic response in polymer networks.

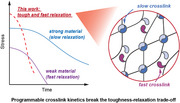

## 1 Introduction

**FIGURE 1:**
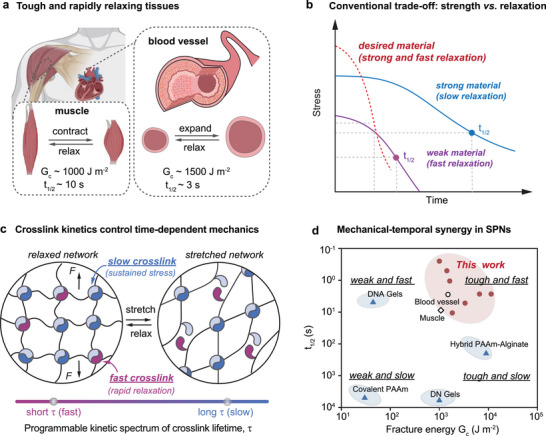
Kinetic control of crosslink dynamics enables mechanical–temporal synergy in SPNs. (a) Native load‐bearing tissues such as muscle and blood vessels combine high fracture energy ($G_{c}∼1000$–1500 J $m^{-2}$) with rapid stress relaxation ($t_{1/2}$ ∼ 3–10 s) [18, 19, 20]. (b) Synthetic hydrogels typically exhibit a trade‐off between strength and relaxation, where fast‐relaxing materials are weak and strong materials relax slowly ($t_{1/2}$). (c) Programmable crosslink lifetimes govern network strength and relaxation dynamics in SPNs. (d) SPNs achieve mechanical–temporal synergy distinct from conventional hydrogels and comparable to native tissues. See Table S1 for quantitative benchmark values and references.

Synthetic hydrogels capable of combining high mechanical modulus, toughness with rapid viscoelastic stress relaxation are critical for biomedical applications such as artificial cartilage, muscle, and wound healing [1, 2]. Native soft tissues inherently integrate these properties, enabling them to sustain repeated mechanical loading while rapidly dissipating stress and adapting to dynamic physiological environments [3, 4, 5]. For example, blood vessels and muscles exhibit rapid stress relaxation (time for stress to decay to half its initial value, $t_{1/2}$ ∼1–10 s) while maintaining high modulus and fracture toughness ($G_{c}$ ∼1000–1500 J $m^{-2}$) under cyclic stress (Figure 1a).

In polymer networks, long‐lived crosslinks govern modulus and network integrity, whereas high fracture energy typically requires sacrificial or reversible dissipation. Many strategies have been developed to toughen synthetic hydrogels, including double‐network designs, nanofillers, and chain entanglement, achieving $G_{c}$ $∼10^{3}$–$10^{4}$ J $m^{-2}$ [6, 7, 8, 9, 10, 11]. However, these materials predominantly rely on densely crosslinked or multi‐network structures that suppress stress relaxation ($t_{1/2}$ > hundreds of seconds) and ultimately limit viscoelastic adaptability under physiological conditions. Alternatively, dynamic covalent or supramolecular chemistries, such as hydrazone exchange or metal–ligand coordination, offer viscoelastic behavior through reversible bonding. While the relaxation kinetics of these systems can be widely tuned through chemical or environmental control (e.g., pH or catalysts), achieving rapid stress relaxation without compromising mechanical strength under load‐bearing conditions remains challenging [12, 13, 14, 15, 16, 17]. Consequently, synthetic hydrogels remain confined to a mechanical–temporal trade‐off: strong networks relax slowly, whereas rapidly relaxing networks are fragile (Figure 1b) [2].

**FIGURE 2:**
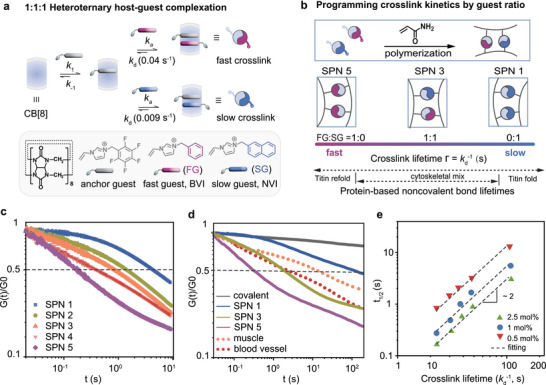
Design principle of SPNs with programmable stress relaxation. (a) CB[8]‐mediated 1:1:1 complexation between an anchor guest (5FBVI) and either a fast guest (FG) – BVI, or a slow guest (SG) – NVI. (b) Mixing FG and SG in controlled ratios (0:1, 1:2, 1:1, 2:1, 1:0) tunes the crosslink lifetime ($\tau =k_{d}^{-1}$) of SPN 1–5 across a protein‐like kinetic spectrum. (c) Normalized stress relaxation curves of SPNs. (d) Comparison with covalent PAAm hydrogels, blood vessels, and muscle. (e) Relaxation half‐time ($t_{1/2}$) scales approximately quadratically with crosslink lifetime ($k_{d}^{-1}$), with a fitted exponent of ∼1.96 and $R^{2}=0.96$–0.97 across crosslinking densities.

To overcome this limitation, we introduce a *kinetic‐programming* framework that functionally decouples relaxation dynamics from modulus and toughness within a single polymer network by encoding crosslink lifetime as a molecular design variable. As illustrated in Figure 1c, supramolecular polymer networks (SPNs) with reversible crosslinks enable precise tuning of crosslink lifetimes across a broad kinetic spectrum comparable to protein‐like noncovalent interactions. Here, the term kinetic programming is used to describe the encoding of supramolecular crosslink lifetimes as a molecular design variable for controlling time‐dependent mechanics, which is achieved through stoichiometry rather than chemical modification. We implement this principle using cucurbit[8]uril (CB[8])–mediated host–guest chemistry, a robust and modular platform that affords systematic control of guest dissociation rates (Figure 2a,b). Leveraging this orthogonal kinetic control, our approach allows synthetic hydrogels to reproduce the mechanical‐temporal synergy of native tissues, accessing the previously inaccessible **tough‐yet‐rapidly relaxing** regime (Figure 1d). More broadly, unlike previous strategies that primarily tune network architecture or polymer chemistry, the kinetic‐programming framework provides a general molecular route to time‐programmable soft materials for biointegrated devices and soft robotics.

## 2 Kinetic Programming of Crosslink Lifetimes Governs Time‐Dependent Mechanics

We employed a series of guest molecules exhibiting polar–π interactions with cucurbit[8]uril (CB[8]) to form dynamic crosslinking motifs with high binding strength (Figure 2a), yielding tunable viscoelastic SPNs with exceptional compressive strength [21]. The supramolecular motif consists of an anchor guest (*N*‐vinylimidazolium derivative bearing a perfluorophenyl group, 5FBVI) that first forms a stable 1:1 binary complex with CB[8]. Driven by electrostatic repulsion, the system then preferentially binds an electron‐rich fast (phenyl, BVI) or slow (naphthyl, NVI) guest, producing an exclusive 1:1:1 heteroternary complex. The structure of the second guest (BVI or NVI) governs the viscoelastic modulus of the SPNs. Isothermal titration calorimetry (ITC) revealed guest‐dependent differences in dissociation rate constant $k_{d}$ (Figure 2a). We hypothesized that varying the ratio of fast to slow guests (BVI:NVI) would allow programmable modulation of crosslink lifetimes ($\tau \;=\;k_{d}^{-1}$, in seconds) and thus tune the network's relaxation behavior [22, 23, 24]. To validate this concept, supramolecular crosslinkers with varying fast: slow guest ratios (0:1, 1:2, 1:1, 2:1, 1:0) were first preassembled through the exclusive 1:1:1 host–guest recognition mechanism (Figure 2b), and ITC measurements confirmed systematic modulation of the corresponding dissociation rates $k_{d}$ (Figures S2 and S3). SPNs 1–5 were then synthesized by photo‐initiated copolymerization of these supramolecular crosslinkers with acrylamide monomers (Figure 2b and Figure S4), providing precise kinetic control through molecular design. Remarkably, the accessible τ range (∼10–200  s) is comparable to the lifetimes of protein‐based noncovalent crosslinks, such as titin unfolding and cytoskeletal bond refolding [25], underscoring the biomimetic nature of this molecular design. The resulting dynamic networks dissipate energy under strain and self‐recover upon unloading, enabled by reversible association and dissociation of the host‐guest complexes. This supramolecular architecture provides tunable, time‐dependent mechanical properties through direct control of crosslink kinetics, bridging molecular dynamics with tissue‐like mechanical behavior.

## 3 Regulating Stress Relaxation by Crosslink Dissociation Kinetics

Having established kinetic control at the molecular level, we next examined how programmed crosslink lifetimes manifest in macroscopic stress‐relaxation behavior–an essential property for biomaterial performance. Transient‐mode rheology was performed under fixed strain while monitoring stress decay over time. At a crosslinking density of X=1 mol%, SPN 5 containing only the fast guest exhibited rapid stress decay (Figure 2c, purple curve), whereas SPN 1 containing only the slow guest relaxed more slowly (Figure 2c, blue curve). By adjusting the fast:slow guest ratio, the stress–relaxation rate is finely tunable: the relaxation half‐time $t_{1/2}$ (dashed line) increases systematically with the fraction of slow guest. Notably, $t_{1/2}$ values of SPNs could be readily controlled over at least three orders of magnitude, from 0.1 s up to 100 s (Figure 2d), encompassing the physiological relaxation of blood vessels ($t_{1/2}$ = 2.6 s) and muscle ($t_{1/2}$ = 10 s), both materials that combine rapid stress dissipation with mechanical strength. Importantly, SPNs preserve their modulus and fast stress‐relaxation behavior during long‐term immersion in physiologically relevant PBS (Figure S5). Dynamic covalent chemistries such as hydrazone exchange, however, typically possess much longer lifetimes ($k_{d}^{-1}∼10^{2}$–$10^{4}$ s) [12], making such rapid relaxation inaccessible. In contrast, covalently crosslinked PAAm gels exhibited negligible relaxation ($t_{1/2}$ >1000 s, Figure 2d), underscoring the decisive role of programmable crosslink kinetics.

To directly link macroscopic relaxation with molecular kinetics, we correlated the relaxation half‐time ($t_{1/2}$) with the measured crosslink lifetime ($\tau =k_{d}^{-1}$) for SPN 1–5 (Figure 2e). Increasing the density of dynamic crosslinkers further accelerates stress relaxation, arising from more frequent exchange among neighboring host–guest complexes that facilitates network reconfiguration (Figure S6) [26]. A quadratic scaling relationship between $t_{1/2}$ and $k_{d}$ 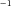 was found for SPN 1–5, which holds across various crosslinking densities (0.5, 1 and 2.5 mol%) for all SPNs. The near‐quadratic scaling (slope ∼1.96) reveals that the network's relaxation dynamics is quantitatively governed by the crosslink dissociation lifetime. It further reflects the cooperative nature of network rearrangement, in which stress relaxation proceeds through multi‐step dissociation‐reassociation events rather than isolated bond‐breaking (Figure S9 and subsequent discussion for details). To verify that this kinetic scaling is general rather than chemistry‐specific, we synthesized an additional SPN using a distinct guest (1‐(4‐chlorobenzyl)‐3‐vinylimidazolium bromide (ClBVI), $k_{d}$ ∼ 0.031 $s^{-1}$), equivalent to a 3:2 ratio of fast:slow guest. Its relaxation behavior ($t_{1/2}$ ∼0.8 s) aligned with the established scaling law (Figure S9). This result showcases how precise molecular control can be leveraged to engineer macroscopic properties, enabling the direct programming of stress relaxation in polymer networks by tuning crosslink kinetics.

## 4 Tuneable Modulus and Viscoelasticity

**FIGURE 3:**
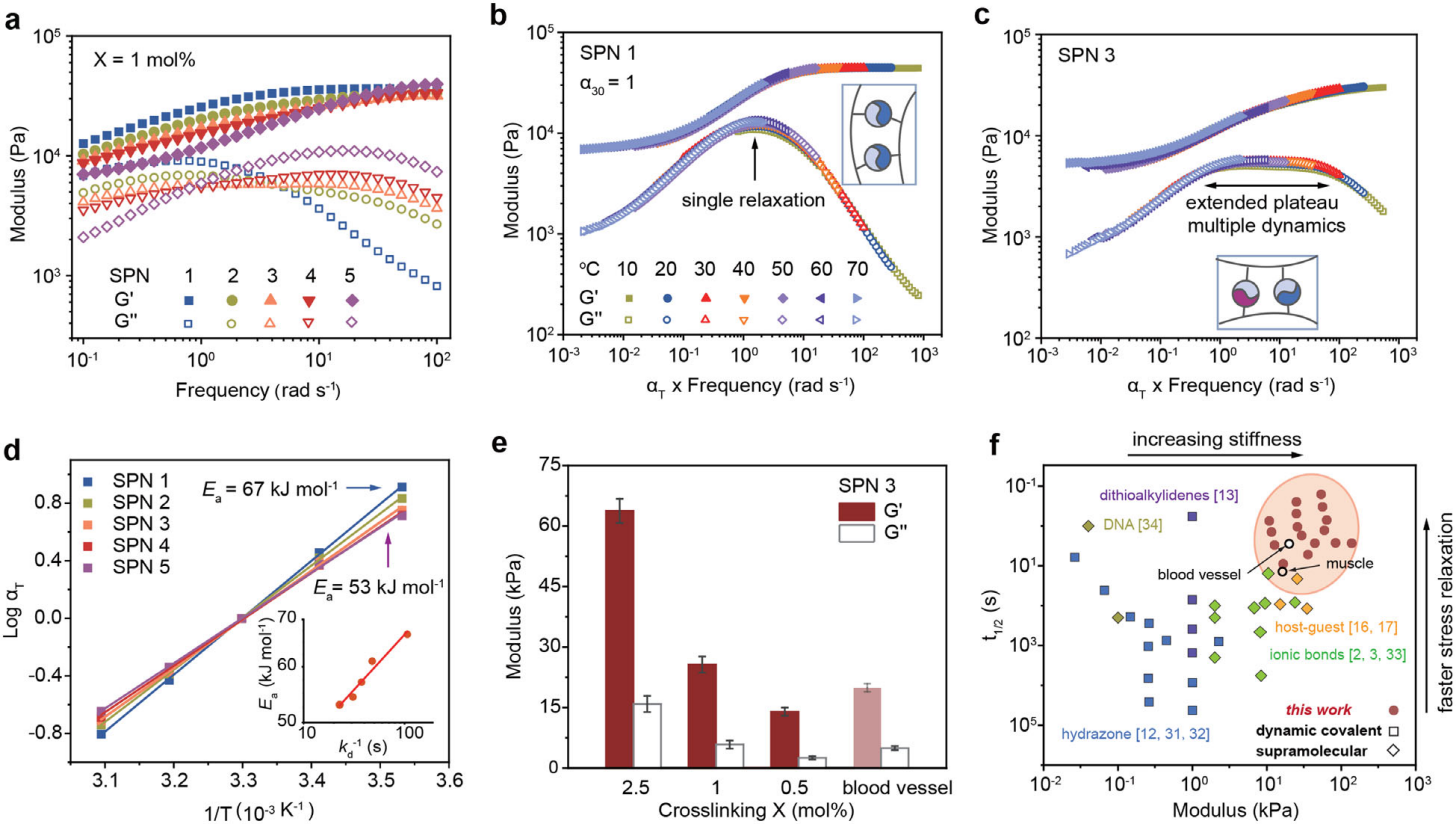
Rheological characterization revealing dynamic crosslink–governed viscoelasticity in SPNs. (a) Frequency sweeps for SPNs 1–5 (T = 20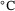). (b) Master curve of SPN 1 exhibiting single relaxation dynamics. (c) Master curve of SPN 3 showing an extended plateau and multiple dynamics. (d) Arrhenius plots of shift factors $\alpha_{T}$ with activation energies $E_{a}$ (inset: $E_{a}$ vs. crosslink lifetime $k_{d}^{-1}$). (e) Storage ($G^{\prime }$) and loss ($G^{\prime \prime }$) moduli of SPN 3 at different crosslinking densities compared with a blood vessel reference. (f) Benchmark of stress relaxation half‐time ($t_{1/2}$) vs. elastic modulus for representative soft material systems including those mediated by dynamic covalent and supramolecular chemistries. Numbers in brackets denote references.

The linear viscoelastic behavior of SPNs was examined by oscillatory rheology (Figure 3a). Across all samples, the storage modulus ($G^{\prime }$) exceeded the loss modulus ($G^{\prime \prime }$), confirming gel‐like behavior. The dependence of $G^{\prime }$ and $G^{\prime \prime }$ to applied frequency reflects the dynamic nature of the SPNs. SPN 1 exhibited the highest $G^{\prime }$, owing to slower crosslink kinetics that allow more bonds to remain intact during deformation. In contrast, the fast‐dissociating crosslinks in SPN 5 are able to decomplex and recomplex readily with neighboring complexes on short time scales, resulting in enhanced viscoelasticity. At high frequency, SPN 1 demonstrated a loss factor of tanδ≈0.02, an order of magnitude lower than SPN 5 (tanδ≈0.2), indicating glass‐like and rubber‐like responses, respectively (Figure S10). Controlling the ratio of fast and slow guests allows direct modulation of viscoelasticity between rubber‐like and glass‐like responses.

To elucidate the origin of rapid stress relaxation in the SPNs, frequency sweeps combined with time–temperature superposition (TTS) were used to extend the accessible frequency range (Figure 3b,c, Figure S12). SPN 1 exhibits a single relaxation mode, manifested as a distinct peak in $G^{\prime \prime }$ whereas SPN 3 displayed an extended $G^{\prime \prime }$ plateau, indicative of multiple dynamic relaxation processes arising from mixed crosslink kinetics. Such broad, frequency‐independent loss moduli are characteristic of polymer networks containing bonds with a spectrum of relaxation times, as described by classical models of transient networks and associative polymers [26]. Although each network can be described by an effective relaxation half‐time ($t_{1/2}$), the broad $G^{\prime \prime }$ plateau indicates a spectrum of network dynamics governed by the coexistence of fast and slow host–guest interactions. Plotting the temperature dependence of the shift factor α 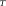 (Figure 3d) enables calculation of the activation energy ($E_{a}$) from the Arrhenius equation α 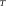 = A $e^{\frac{E_{a}}{RT}}$, where α 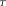 is the horizontal shift factor, and A is a pre‐exponential constant. $E_{a}$ values for SPN 1–5 ranged from 53–67 kJ ${\mathrm{mol}}^{-1}$ (13–16 kcal ${\mathrm{mol}}^{-1}$), which scale with crosslink lifetime ($k_{d}^{-1}$; slope ≈0.16). The higher $E_{a}$ values of the slowest SPNs agree with their slower stress relaxation (Figure 2c), confirming that energy barriers for bond dissociation directly govern macroscopic dynamics. These activation energies are comparable to the unfolding barrier of the titin I27 domain (17 kcal ${\mathrm{mol}}^{-1}$) [27], yet remain far below those of covalent bonds ($E_{C-C}$ ≈347 kJ ${\mathrm{mol}}^{-1}$), or hydrogen‐ ($E_{a}$ ∼178–259 kJ ${\mathrm{mol}}^{-1}$) and ionically bonded networks ($E_{a}$ ∼248–308 kJ ${\mathrm{mol}}^{-1}$) [28, 29, 30]. The relatively low $E_{a}$ values imparted by the dynamic supramolecular crosslinks facilitate preferential bond dissociation under deformation, thereby enabling the rapid stress relaxation absent in conventional hydrogels.

To probe how network architecture influences viscoelasticity, SPN 3 was examined at varying crosslink densities (X = 0.5–2.5 mol%) and monomer concentrations ($C_{M}$ = 1–4 M) (Figure 3e). Increasing X raised the storage modulus ($G^{\prime }$) from 10 to 60 kPa, encompassing the stiffness of blood vessels (∼20 kPa), while variations in $C_{M}$ enhanced $G^{\prime }$ without altering the relaxation half‐time ($t_{1/2}$) as evidenced by the overlap of normalized stress‐relaxation curves (Figure S7). These results indicate that relaxation behavior is governed primarily by the kinetics of supramolecular complexes, rather than by polymer density alone, enabling orthogonal control over modulus and relaxation. Compared with dynamic covalent [12, 13, 31, 32] and supramolecular networks [2, 3, 16, 17, 33, 34, 35], the SPNs represent a kinetically programmable platform that enables simultaneous tuning of stiffness and stress relaxation within a single network chemistry, overlapping with the mechanical regime of load‐bearing tissues (Figure 3f).

## 5 Crosslink Kinetic‐Controlled Fracture Toughness

**FIGURE 4:**
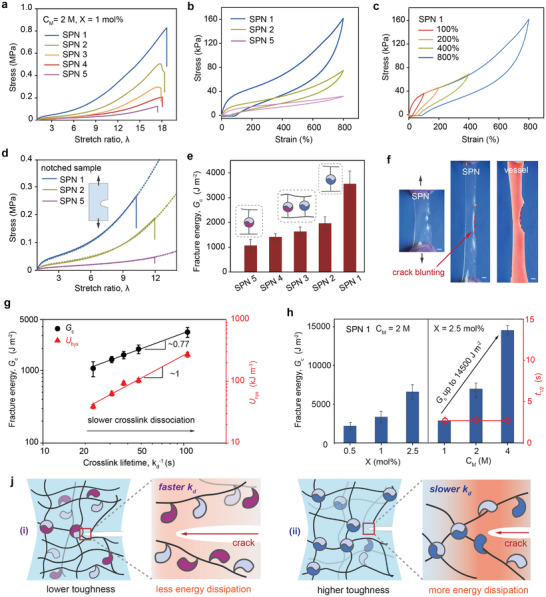
Kinetic control of mechanical performance. (a) Uniaxial stress–strain curves of SPNs. (b) Hysteresis behavior showing enhanced energy dissipation with slower crosslink kinetics. (c) Consecutive loading–unloading cycles demonstrating full recovery. (d) Stress–strain curves of notched SPNs (dashed line, unnotched). (e) Fracture energy, $G_{c}$. (f) SPNs and blood vessel showing crack blunting (scale bar is 3 mm). (g) Scaling of $G_{c}$ and dissipated energy ($U_{\mathrm{hys}}$) with crosslink lifetime ($k_{d}^{-1}$). Power‐law fits yield exponents of 0.77 for $G_{c}$ ($R^{2}=0.99$) and 1.02 for $U_{\mathrm{hys}}$ ($R^{2}=0.97$). (h) Independent tuning of $G_{c}$ and $t_{1/2}$ by crosslink density and monomer concentration. (j) Schematic fracture mechanisms for (i) fast‐ and (ii) slow‐dissociating crosslinks.

Building on these rheological insights into rapid stress relaxation, we next examined how crosslink kinetics influence large‐deformation and fracture behavior. Figure 4a displays that all SPNs tested exhibited high stretchability with a critical stretch ratio λ 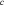 up to ∼ 18. As the proportion of slow guest increased, the rupture stress improved 8‐fold, implying that slow dissociation of crosslinks contributes to higher mechanical strength. Notably, As expected for dynamically crosslinked networks, the tensile behavior of the SPNs showed strong dependence on strain rate (Figure S13), consistent with the observed correlation between frequency and moduli discussed above (Figure 3a). Cyclic loading–unloading tests further revealed that slower crosslink kinetics enhance energy dissipation (Figure 4b). SPN 1 exhibited a large hysteresis loop ($U_{\mathrm{hys}}=0.27\;\mathrm{MJ},m^{-3}$), threefold higher than SPN 2 and nearly sevenfold higher than SPN 5. Although transient residual strain is observed immediately upon unloading, particularly in samples with slower crosslink kinetics, this behavior reflects the finite exchange kinetics of supramolecular bonds rather than irreversible deformation. All SPNs showed full self‐recovery within 2 min (Figure 4c and Figure S14), arising from the reversible association and dissociation processes of the host‐guest crosslinks. Subsequently, single‐edge notch tests were conducted on SPN specimens to evaluate fracture toughness. The stress–strain curves for notched SPNs (Figure 4d) show that SPN 1 has both a higher critical strength and lower critical strain for crack propagation compared to SPNs with a larger fast guest content. Fracture energy ($G_{c}$) was calculated for SPN 1–5 (X = 1 mol%, $C_{M}$ = 2 M) and the results (Figure 4e) show values ranging from 1100 to 3340 J $m^{-2}$. Under large deformation, SPN 1 displayed pronounced crack blunting (Figure 4f), analogous to that observed in blood vessels, a hallmark of tough soft materials. Additionally, an intriguing crack‐deflection phenomenon was frequently observed, in which a secondary crack propagated along the stretch direction, deviating from the pre‐cut path (Figures S15 and S16), indicative of enhanced toughness in soft networks [8, 36, 37, 38, 39, 40].

To directly link toughening to supramolecular kinetics, we plotted the fracture toughness ($G_{c}$) and dissipated energy ($U_{\text{hys}}$) as functions of the crosslink lifetime ($k_{d}^{-1}$), revealing a power‐law scaling with exponents of 0.77 and approximately 1, respectively (Figure 4g). This correlation demonstrates that longer‐lived crosslinks enhance both toughness and dissipation. Within the fracture‐energy balance framework [41, 42, 43, 44]. $G_{c}∼U_{\text{hys}}\;l_{\text{eff}}$, we define an effective energetic coupling length $l_{\text{eff}}∼G_{c}/U_{\text{hys}}$, which exhibits a weak dependence on crosslink lifetime, $l_{\text{eff}}∼\tau^{-0.23}$, with $\tau =k_{d}^{-1}$. This phenomenological metric captures how efficiently bulk hysteretic dissipation contributes to fracture resistance, with the trend suggesting that slower crosslink dynamics are associated with higher bulk dissipation and more localized deformation near the crack tip. Digital image correlation (DIC) measurements (Figure S17) are used as a qualitative probe of crack‐tip strain fields in SPNs, consistent with the phenomenological scaling trends. Energy dissipation has also been visualized in mechanophore‐tagged multiple‐network elastomers [45, 46], and the present system further enables molecular control via crosslink kinetics.

Building on this kinetic insight, we clarify how crosslink dynamics govern toughening in SPNs. When a gel containing a crack is stretched, deformation is inherently inhomogeneous, with the region ahead of the crack experiencing the highest strain compared to that applied to the whole sample [42]. Two fundamental strategies for improving fracture resistance are (i) reducing stress concentration at the crack front (e.g. crack blunting and crack deflection), and (ii) introducing efficient energy dissipation mechanisms (e.g. dynamic molecular crosslinks or introducing a secondary network to form double network gels) [45, 47, 48, 49]. In covalently crosslinked PAAm, $G_{c}$ is low (26 J $m^{-2}$; Figure S18) because only a small fraction of chains dissipate energy. In SPNs with faster‐dissociating crosslinks (Figure 4j(i)), reversible host–guest interactions act as sacrificial bonds that break and reform during deformation, contributing to energy dissipation. SPNs with slower‐dissociating crosslinks (Figure 4j(ii)) undergo gradual unzipping of the network, where long‐lived crosslinks dissipate more energy per event yet within a smaller dissipation zone. This confined, energy‐dense dissipation stabilizes deformation and slows crack advance, in contrast to the brittle, energy‐poor localization in covalent gels.

Extending this concept, tuning crosslink density and polymer concentration enables access to tough yet dynamic hydrogels with exceptional fracture toughness (Figure 4h, Figures S19 and S20). For SPN 1, increasing the crosslinking density from 0.5–2.5 mol% ($C_{M}$ = 2 M) led to a substantial rise in $G_{c}$ from 2150 to 6570 J $m^{-2}$. At the fixed crosslinking density X = 2.5 mol%, the toughness could be further enhanced by increasing the monomer concentration from 1 to 4 M, reaching a $G_{c}$ value of 14 500 J $m^{-2}$, primarily attributed to additional polymer chain entanglements that contribute to energy dissipation during fracture. Increasing monomer concentration also increases the polymer volume fraction of the final gel and correspondingly reduces its water content resulting in a denser load‐bearing network. Table S2 summarizes the water content and fracture energy of SPNs in comparison to representative tough soft material systems. The toughness of SPNs in this work exceeds that of many double‐network hydrogels ($G_{c}$ ∼ 1000 J $m^{-2}$) [50]. biological tissues such as cartilage, muscle and blood vessels ($G_{c}$ ∼ 1000 – 2000 J $m^{-2}$) [51]. and even natural rubbers ($G_{c}$ ∼ 10 000 J $m^{-2}$) [52]. Increasing crosslink density at fixed $C_{M}$ strengthens the network while simultaneously accelerating stress relaxation (Figure S6), indicating that relaxation is governed by supramolecular exchange kinetics rather than network topology. In contrast, at fixed crosslink density, the stress–relaxation time $t_{1/2}$ remains essentially invariant across the $C_{M}$ range (Figure 4h), even as $G_{c}$ increases by nearly an order of magnitude. Moreover, entanglement‐controlled physical PAAm gels without supramolecular crosslinks exhibit markedly slower relaxation (Figures S6– S8), underscoring the distinct role of host‐guest bond exchange in governing relaxation in SPNs. Consistent with this perspective, network imperfections such as dangling or inactive chains have little influence on the relaxation half‐time $t_{1/2}$ (see Figure S8 for further discussion). Together, these trends demonstrate a functional decoupling of toughness and relaxation: monomer concentration primarily controls the energy‐dissipation capacity of the network, whereas the characteristic relaxation time is set by the host‐guest dissociation kinetics. This orthogonal control enables mechanical strengthening without sacrificing rapid stress relaxation.

## 6 Conclusion

By encoding programmable lifetimes into supramolecular crosslinks, we show that molecular dissociation kinetics can directly dictate macroscopic mechanical response, beyond conventional control via polymer structure or composition. This establishes a kinetic programming strategy for designing soft materials in which fracture toughness and stress relaxation are controlled via supramolecular bond dynamics. The resulting networks access rapid stress relaxation ($t_{1/2}$ = 0.1–100 s) while achieving high fracture energy ($G_{c}$ > 14 000 J $m^{-2}$), overcoming the mechanical–temporal trade‐off in synthetic hydrogels. As the supramolecular crosslinkers are vinyl‐terminated and compatible with a broad range of radical–polymerizable monomers (including zwitterionic, thermoresponsive, conductive, and UV‐curable chemistries) this kinetic‐programming concept is readily transferable to diverse hydrogel platforms and soft–matter systems. Looking forward, incorporating multiple or stimuli‐responsive kinetic motifs could endow materials with spatiotemporally adaptive mechanics, enabling real‐time reconfiguration under mechanical or environmental cues. Extending kinetic programming to heterogeneous or gradient architectures may further localize energy dissipation and relaxation, mirroring the behavior of biological tissues. Rather than relying on structural hierarchy, as in double‐network or nanocomposite gels, this work highlights crosslink lifetime as a tunable molecular handle for programming viscoelastic response in soft materials. Together, these findings support kinetic control of supramolecular dissociation as a viable strategy for engineering energy‐dissipative and adaptive hydrogels whose mechanical behavior is governed at the molecular level.

## 7 Experimental Section

### 7.1 Materials

Unless otherwise stated, all the chemicals in this research were purchased from Sigma–Aldrich and used without further purification: acrylamide (for molecular biology, 99%, HPLC), 1‐vinylimidazole (99%), 2,3,4,5,6‐perfluorobenzyl bromide (99%), benzyl bromide (reagent grade, 98%), 2‐(bromomethyl) naphthalene (96%), 4‐chlorobenzyl bromide (97%), *N*‐hydroxyethyl acrylamide (97%, 1000 ppm MEHQ), acetonitrile (HPLC, 99.9%), acetone (ACS reagent, 99.5%), diethyl ether (ACS reagent, 99%), deuterium oxide ($D_{2}O$, D 99.8 atom%), nitrogen, 2‐Hydroxy‐4'‐(2‐hydroxyethoxy)‐2‐methylpropiophenone (I‐2959, photoinitiator, 98%), and *N,N'*‐Methylenebisacrylamide (MBA).

Cucurbit[8]uril (CB[8]) was synthesised and isolated on a 100‐gram scale from the mixture of cucurbit[n]uril (CB[n]) derivatives using a previously reported protocol [53, 54]. All the guest molecules were readily synthesized through salt formation reactions between various substituted benzyl bromides and 1‐vinylimidazole, following the same procedure that we have previously reported [21, 55].

Details of synthesis of 1‐(2,3,4,5,6‐perfluorobenzyl)‐3‐vinylimidazolium bromide (5FBVI), 1‐(2‐Naphthylmethyl)‐3‐vinylimidazolium bromide (NVI), 1‐Benzyl‐3‐vinylimidazolium bromide (BVI), 1‐(4‐Chlorobenzyl)‐3‐vinylimidazolium bromide (ClBVI) are in the Supporting Information.

### 7.2 Methods

#### 7.2.1 Procedures for Preparation of Supramolecular Polymer Network Hydrogels (SPNs)

SPNs were prepared as our previous literature reports [21, 55, 56, 57] and detailed in the Supporting Information.

#### 7.2.2 Nuclear Magnetic Resonance (NMR) Spectroscopy


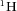
 NMR spectra were acquired in $D_{2}O$ at 298.15 K on a Bruker AVANCE 500 with TCI Cryoprobe system (500 MHz) being controlled by TopSpin2. Chemical shifts for proton peaks in 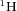 NMR were referenced to the residual solvent peak (HDO) at 4.79 ppm.

#### 7.2.3 Isothermal Titration Calorimetry (ITC)

ITC measurements were performed using a *Malvern MicroCal Auto‐ITC‐200* instrument at 298.15K in Milli‐Q $H_{2}O$. For the second binding event, the 1:1 host–guest complex (5FBVI–CB[8]) was loaded into the sample cell at 0.5mM, and the second guest was loaded in the syringe at a tenfold higher concentration of 5.0mM. Each titration consisted of one initial injection of 0.6μL followed by 32 injections of 1.2μL, with 90s intervals between injections. The first one or two data points were discarded prior to analysis due to potential artefacts. Resulting ITC isotherms were fitted with a sequential binding model in the *Malvern MicroCal Analysis Centre* software to obtain thermodynamic parameters for the second binding step. The ITC data were further analysed using the kinITC method  [58] in *Affinimeter‐ITC‐Advanced* to extract association and dissociation rate constants for all second‐guest binding processes. All titrations were repeated three times to obtain mean thermodynamic and kinetic parameters with corresponding standard deviations (n=3).

#### 7.2.4 Rheology

Rheological characterization was implemented by a Discovery Hybrid Rheometer (DHR‐2, TA Instruments) with a Peltier Plate for temperature control. All measurements were conducted using a 20 mm parallel stainless steel plate geometry with a fixed gap of 2000 μm, and necessary calibration for geometry was carried out before testing. Oscillatory frequency sweep measurements were conducted at 1.0% strain within the linear viscoelastic region in the frequency range from 0.1 to 100 rad $s^{-1}$. The above data were collected at 293.15 K and analysed by TRIOS software, TA Instruments.

#### 7.2.5 Stress Relaxation

The tests were performed by applying a constant strain of 5% within 0.01 s and monitoring the resultant instant modulus G(t). Normalised stress relaxation curves, $G\left(t\right)/G_{0}$, are derived by dividing the instant modulus G(t) by the initial modulus of the specimens $G_{0}$.

#### 7.2.6 Time–Temperature Superposition Principle

The shift factors can be correlated with the temperature using the Arrhenius equation as follows:

$$\log\left(\alpha_{T}\right)=\frac{E_{a}}{2.03R}\left(\frac{1}{T}-\frac{1}{T_{\text{ref}}}\right)$$

where $E_{a}$ is the activation energy in kJ ${\mathrm{mol}}^{-1}$ and R is the gas constant (8.314 J $K^{-1}$ ${\mathrm{mol}}^{-1}$). The shift factors can be fitted linearly to the inverse of temperature (1/T). The activation energy is a measure of the energy barrier that must be overcome to allow for the relaxation of the viscoelastic SPNs.

#### 7.2.7 Tensile Test

Uniaxial tensile tests were performed just after the network fabrication, on an Instron (34TM_10) equipped with a 50‐N load cell at room temperature. In a typical tensile test, a dumbbell‐shaped specimen, following ISO4661‐1 standard with dimensions of 10 mm (L) × 2 mm (W) × 2 mm (T), was clamped by grips and stretched at a specific rate (i.e., 50 mm/min) until the break of the specimen. Different strain rates (50, 100, 200 mm/min) were used to study the rate‐effect on tensile behaviors of SPNs.

#### 7.2.8 Cyclic Loading‐Unloading Test

Loading–unloading test was performed for SPNs at a stretch rate of 50 mm/min. Various maximum strain $\varepsilon_{\max}$ was reached to evaluate the self‐recovery properties of SPNs. The waiting time between each loading cycle is 2 min. The energy dissipation $U_{\text{hys}}$ during this loading–unloading process was calculated using the following equation [42]:

$$U_{\text{hys}}=\int_{0}^{\varepsilon_{\max}}\sigma_{\text{load}}\;d\varepsilon -\int_{0}^{\varepsilon_{\max}}\sigma_{\text{unload}}\;d\varepsilon$$

#### 7.2.9 Single‐Edge Notch Fracture Test

A single‐edge notch test was carried out to characterize the fracture energy of the SPNs. The dimensions of specimens are 20 mm (L) × 5 mm (W) × 2 mm (T), with initial crack length 1–1.5 mm made using a razor blade. The notched sample was stretched at a stretching rate of 50 mm/min until the rupture. Fracture toughness $G_{c}$ of the single‐edge notched specimen was calculated using the following equation [42]:

$$G_{c}=\frac{6WC_{0}}{\sqrt{\lambda_{c}}}$$

where $\lambda_{c}$ is the critical fracture stretch ratio when the edge‐notched samples were subjected to uniaxial stretching, W is the strain energy density of an uncracked sample being stretched to the maximum deformation $\lambda_{c}$, and $C_{0}$ is the initial crack length.

## Conflicts of Interest

The authors declare no conflicts of interest.

## Supporting information


**Supporting File**:adma72829‐sup‐0001‐SuppMat.pdf.

## Data Availability

Data generated and analysed during this study are provided as Source Data or included in the Supplementary Information. Further data are available from the corresponding author upon reasonable request.
