# Supplementary material for: Tough and Rapidly Relaxing Hydrogels Via Programmable Crosslink Kinetics
Source: Adv Mater. 2026 Apr 9;38(26):e23440. doi: 10.1002/adma.202523440 (PMC13155329; doi:10.1002/adma.202523440)
Supplement: Supplementary file 1 — Supporting File:adma72829‐sup‐0001‐SuppMat.pdf. [file ADMA-38-e23440-s001.pdf]

Supplementary Information: Tough and Rapidly Relaxing Hydrogels *via* Programmable Crosslink Kinetics

Yuanyuan Wei[1], Stephen J.K. O'Neill[1], Jade A. McCune[1], and Oren A. Scherman*[1]

[1] *Melville Laboratory for Polymer Synthesis, Yusuf Hamied Department of Chemistry, University of Cambridge, Lensfield Road, Cambridge CB2 1EW, UK.*

[*] *e-mail: oas23@cam.ac.uk*

S.1 Synthesis and characterization of guest molecules

Synthesis of 1-(2,3,4,5,6-perfluorobenzyl)-3-vinylimidazolium bromide (5FBVI)

All the guest molecules were readily synthesized through salt formation reactions between various substituted benzyl bromides and 1-vinylimidazole, following the same procedures as we have previously reported.[1,2] As an example, the synthesis of 1-(2,3,4,5,6-perfluorobenzyl)-3-vinylimidazolium bromide (5FBVI) is described below. 2,3,4,5,6-Perfluorobenzyl bromide (20.0 mmol) and 1-vinylimidazole (21.0 mmol) were mixed and dissolved in acetonitrile (30 mL), then heated at 82 °C for 12 h. After the reaction, the mixture was divided into two equal portions in 50 mL Falcon tubes. Diethyl ether (30 mL) was added to each portion to precipitate the imidazolium salt. A white solid was collected by centrifugation at 10,000 rpm for 10 min to remove the supernatant containing excess 1-vinylimidazole. The crude product was washed with 40 mL of diethyl ether in each tube and centrifuged again. Finally, the solid product was combined and dried under vacuum to a constant weight, affording 5FBVI as a white powder in approximately 91% yield. ^{1}H NMR (500 MHz, D_2O, δ): 7.74 (d, 1H), 7.56 - 7.55 (d, 1H), 7.09 - 7.04 (q, 1H), 5.77 -5.73 (q, 1H), 5.58-5.57 (s, 2H), 5.40 - 5.38 (q, 1H).

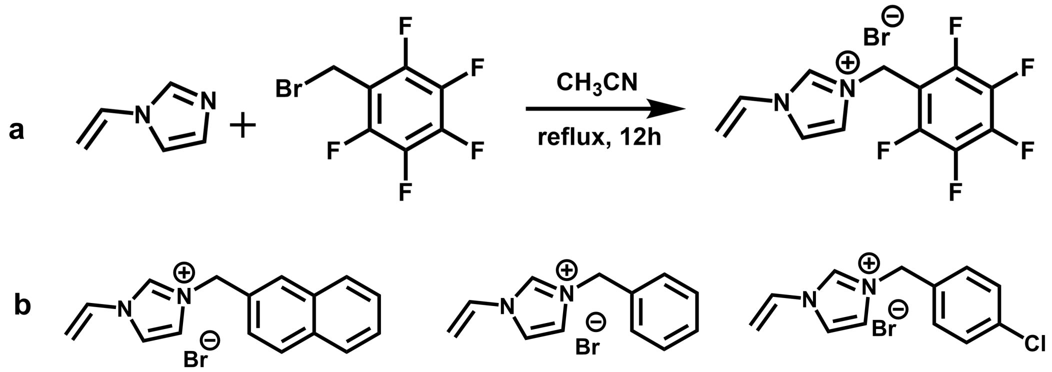

NVI **BVI** **ClBVI**

Supplementary Figure 1. Guest synthesis *via* one-step salt-formation reaction. (a) Representative synthetic route for the preparation of BVI-derivatized guest molecules, illustrated using 5FBVI as an example. (b) Chemical structures of other guest molecules.

Synthesis of 1-(2-Naphthylmethyl)-3-vinylimidazolium bromide (NVI). The same procedure as described for the synthesis of 5FBVI was followed, using 2-(bromomethyl)naphthalene as the starting material. A brown solid was obtained, yielding approximately 88%. ^{1}H NMR (500 MHz, D_2O, δ): 8.92-8.91 (s, 1H), 7.84-7.77 (m, 4H), 7.61-7.60 (d, 1H), 7.50-7.46 (m, 2H), 7.39-7.38 (t, 1H), 7.33-7.31 (q, 1H), 6.98-6.93 (q, 1H), 5.66-5.62 (q, 1H), 5.37 (s, 2H), 5.31-5.29 (q, 1H).

Synthesis of 1-Benzyl-3-vinylimidazolium bromide (BVI). The same procedure as described for the synthesis of 5FBVI was followed, using benzyl bromide as the starting material. A white

solid was obtained, yielding approximately 90%. ^{1}H NMR (500 MHz, D_2O, δ): 8.97-8.98 (s, 1H), 7.71-7.70 (d, 1H), 7.49-7.42 (d, 1H), 7.54-7.36 (m, 5H), 7.06-7.01 (q, 1H), 5.73-5.69 (q, 1H), 5.36-5.33 (m, 3H).

Synthesis of 1-(4-Chlorobenzyl)-3-vinylimidazolium bromide (ClBVI). The same procedure as described for the synthesis of 5FBVI was followed, using 4-chlorobenzyl bromide as the starting material. A white solid was obtained, yielding approximately 96%.^{1}H NMR (500 MHz, D_2O, δ): 8.99-8.98 (s, 1H), 7.72-7.71 (d, 1H), 7.49-7.48 (d, 1H), 7.42-7.31 (m, 4H), 7.07-7.02 (q, 1H), 5.74-5.70 (q, 1H), 5.36-5.34 (m, 3H).

S.2 Characterization of guest binding to CB[8]

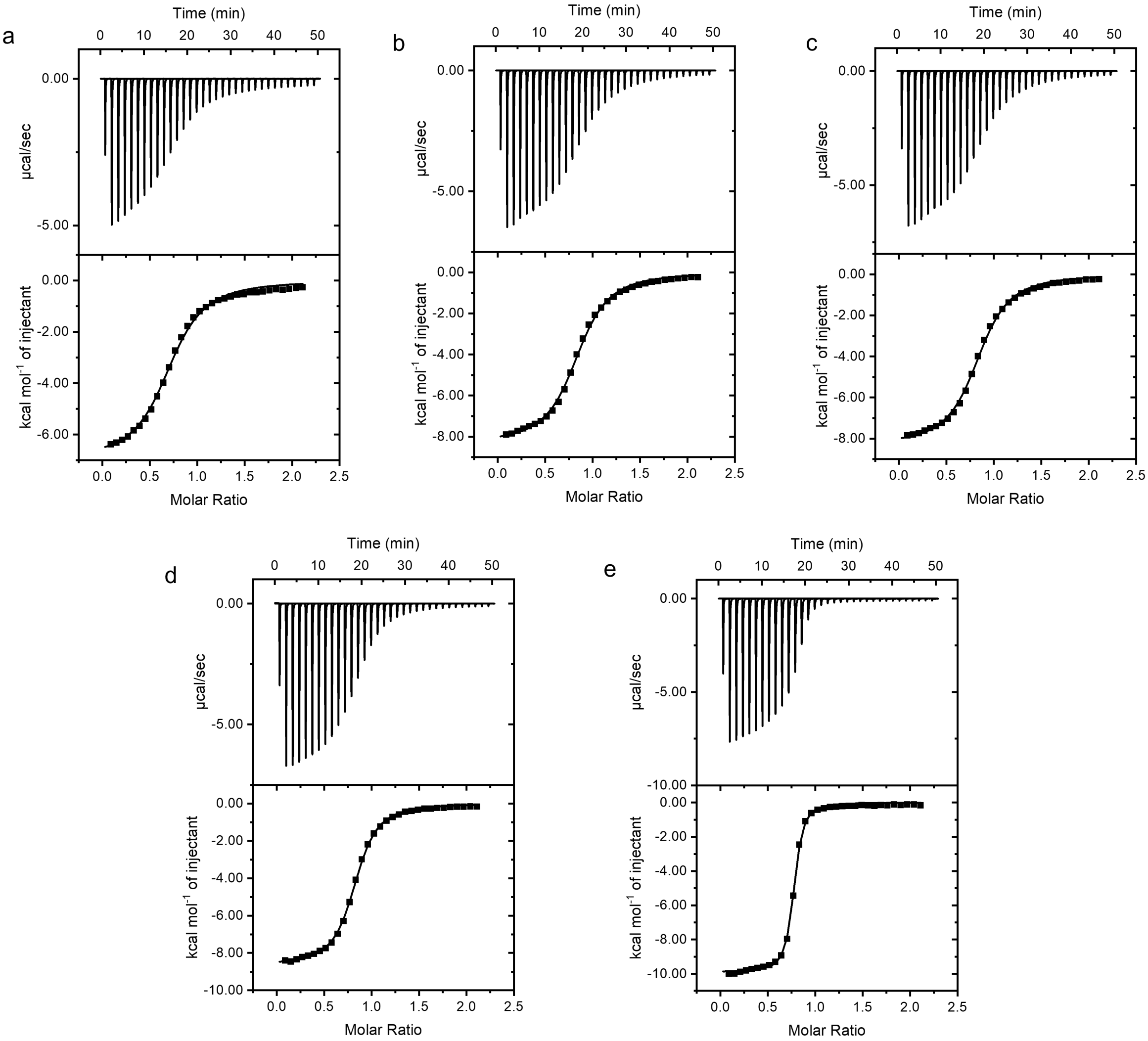

Supplementary Figure 2: Thermodynamic characterization of CB[8]–mediated non-covalent crosslinkers by ITC. Titration traces and fitted curves for injections of (a) fast guest (FG), (b) fast guest : slow guest (FG:SG) = 2:1, (c) FG:SG = 1:1, (d) FG:SG = 1:2, (e) FG:SG = 0:1 into a pre-formed 1:1 5FBVI·CB[8] complex at 298.15 K. Syringe: [Guest] = 5.0 mM; cell: [5FBVI · CB[8]] = 0.50 mM.

As shown in Supplementary Figure 2, ITC titrations of the second guest at FG:SG ratios of 1:0, 2:1, 1:1, 1:2, and 0:1 into the pre-formed 5FBVI·CB[8] complex each display a sharp equivalence point at a 1:1 molar ratio. This stoichiometric breakpoint evidences a strong second-association event and the exclusive formation of 1:1:1 heteroternary complexes, consistent with host–enhanced polar–π interactions within the CB[8] cavity (FG = BVI; SG = NVI).

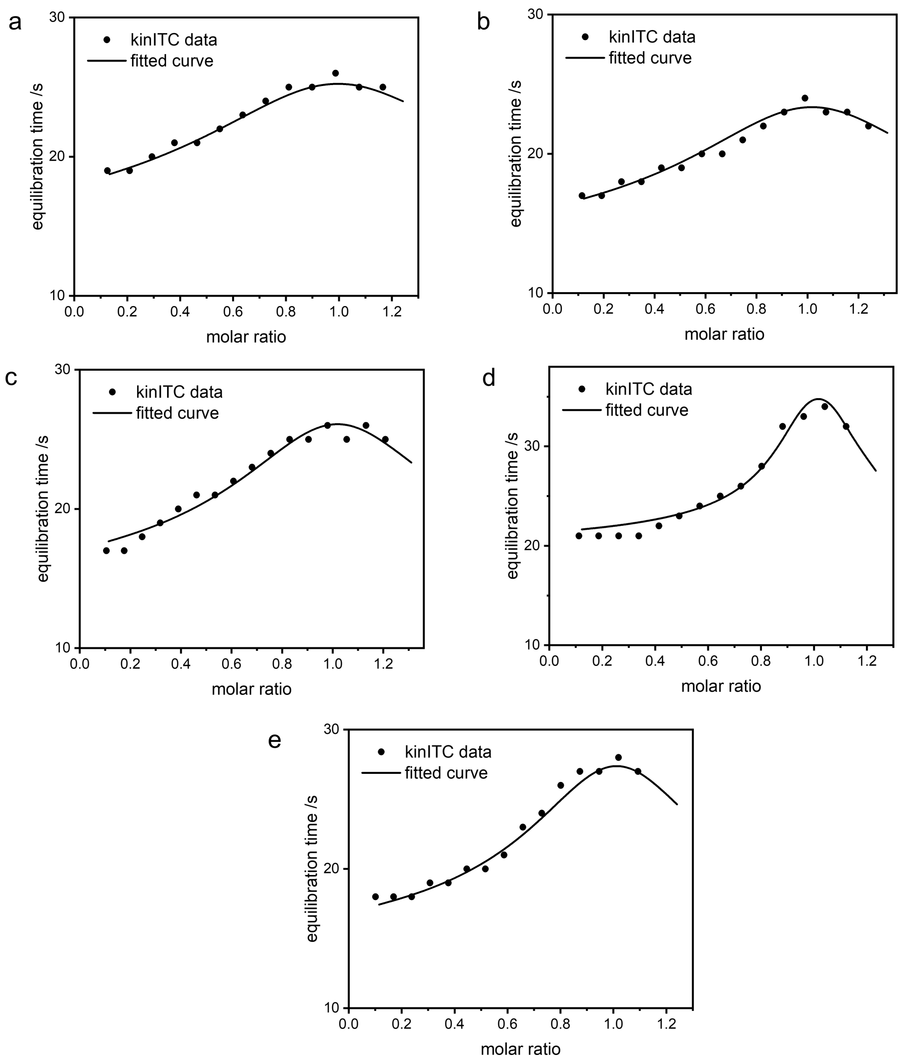

Supplementary Figure 3: Kinetic study of CB[8]–mediated non-covalent crosslinkers. Kinetic ITC (kinITC) plots showing the equilibration time in each titration versus molar ratio, together with fitted curves obtained from ITC titrations of (a) fast guest (FG), (b) fast guest:slow guest (FG:SG) = 2:1, (c) FG:SG = 1:1, (d) FG:SG = 1:2, (e) FG:SG = 0:1 into a 5FBVI–CB[8] complex at 298.15 K.

Analysis of the ITC thermograms (Supplementary Figure 3) provided equilibration times for each injection across the full range of molar ratios. Fitting these data with the kinetic ITC (kinITC) method yielded secondary-association kinetics. In particular, the dissociation rate constants (k_d) for FG:SG compositions of 1:0, 2:1, 1:1, 1:2, and 0:1 were 0.044, 0.032, 0.025, 0.019, and 0.009 s^{-1}, respectively. Thus, the crosslink lifetime $\tau = k_d^{-1}$ is programmably controlled across the series, directly linking molecular kinetics to the time-dependent mechanics of the networks.

S.3 Procedures for preparation of supramolecular polymer network hydrogels (SPNs)

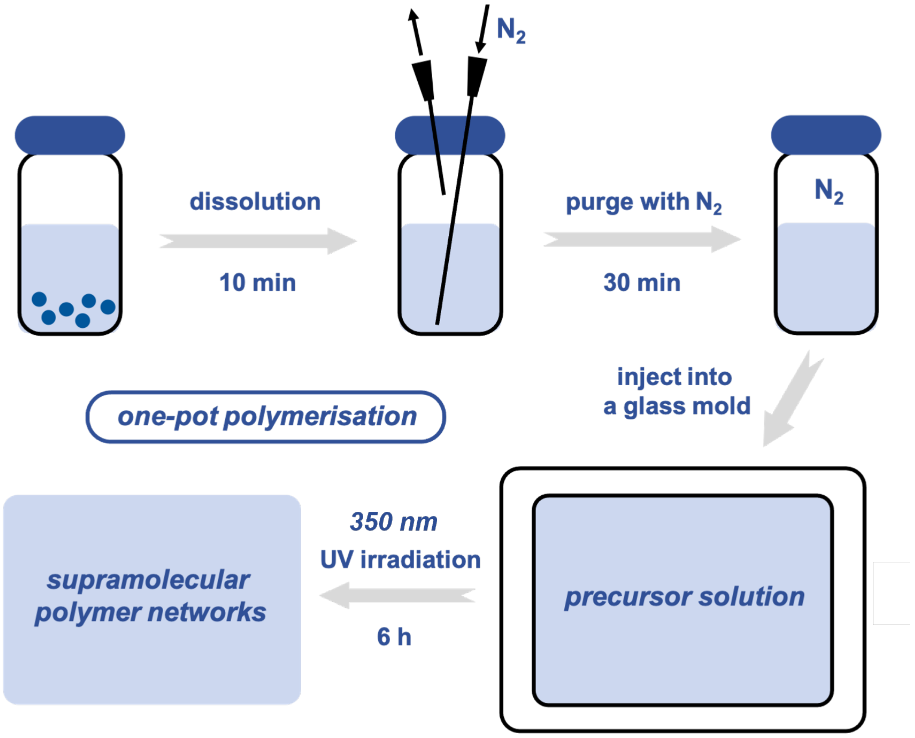

Supplementary Figure 4: One-pot fabrication of supramolecular polymer networks. Schematic representation for the preparation of SPNs in a glass mould.

1) Certain amounts of acrylamide (AAm), non-covalent crosslinker (5FBVI-CB[8]-BVI/NVI), and photoinitiator (I-2959) were pre-determined, weighed out in a glass vial, and dissolved (or dispersed) in a pre-calculated amount of Milli-Q water under ultrasonication for 10 min.
2) The obtained precursor solution was sealed and purged with nitrogen for at least 30 min to remove oxygen in the solution phase that may eliminate radicals during polymerisation.
3) The precursor solution was carefully injected into a laboratory-made glass mould until the whole mould was filled without any bubbles or spare space inside.
4) The glass mould filled with the precursor solution was exposed to UV irradiation at 350 nm with 4.8 mW/cm^2 for 6 h to undergo *in situ* photo-polymerisation in one pot.
5) After *in situ* polymerisation, the SPNs were removed from the glass mould and further cut into the test specimens with different sizes and shapes using a dumbbell/cylinder-shaped cutter or a razor blade.

S.4 Material characterisation of SPNs

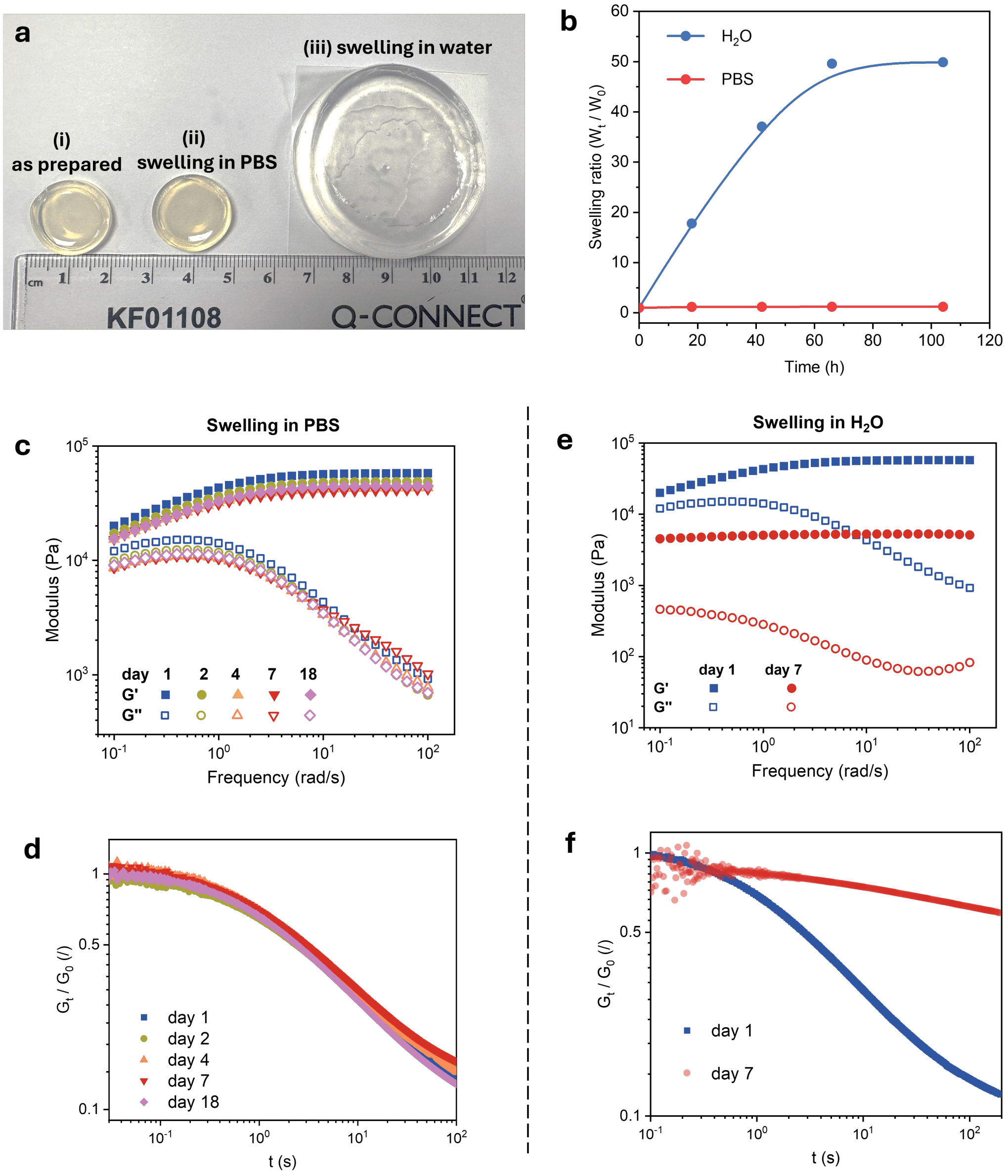

Supplementary Figure S5: Swelling behavior and mechanical evolution of SPNs in water and PBS (SPN 1, $C_M = 2$ M, $X = 2$ mol%). **(a)** Photographs of SPN samples: (i) as-prepared, (ii) after swelling in PBS for 24 h, and (iii) after swelling in deionized water for 24 h. **(b)** Swelling kinetics of SPNs expressed as swelling ratio (sample weight normalized to the as-prepared state) as a function of time. **(c)** Frequency sweep of SPNs after immersion in PBS, showing minimal changes in elastic (G') and viscous (G'') moduli over 18 days. **(d)** Normalized stress relaxation curves of SPNs in PBS. **(e)** Frequency sweep of SPNs after immersion in deionized water. **(f)** Corresponding stress relaxation behavior in deionized water, revealing a marked slowdown of relaxation associated with extensive swelling.

To evaluate the long-term stability of SPNs under physiologically relevant conditions, swelling tests were performed in phosphate-buffered saline (PBS) and deionized water, respectively. As shown in Figure S5a–d, swelling of the SPN networks is strongly suppressed in PBS, where both the elastic and viscous moduli, as well as the stress relaxation dynamics, remain essentially unchanged over 18 days. In contrast, in deionized water the SPNs undergo extensive swelling, reaching an equilibrium swelling ratio of approximately 50 within ~60 h (Figure S5a–b). This pronounced swelling in water leads to a marked decrease in modulus and a significant slowdown of stress relaxation, which is attributed to the dissociation of host–guest crosslinks and dilution of the polymer network. Collectively, these results demonstrate that SPNs retain their mechanical integrity and programmed relaxation behavior under physiologically relevant ionic conditions.

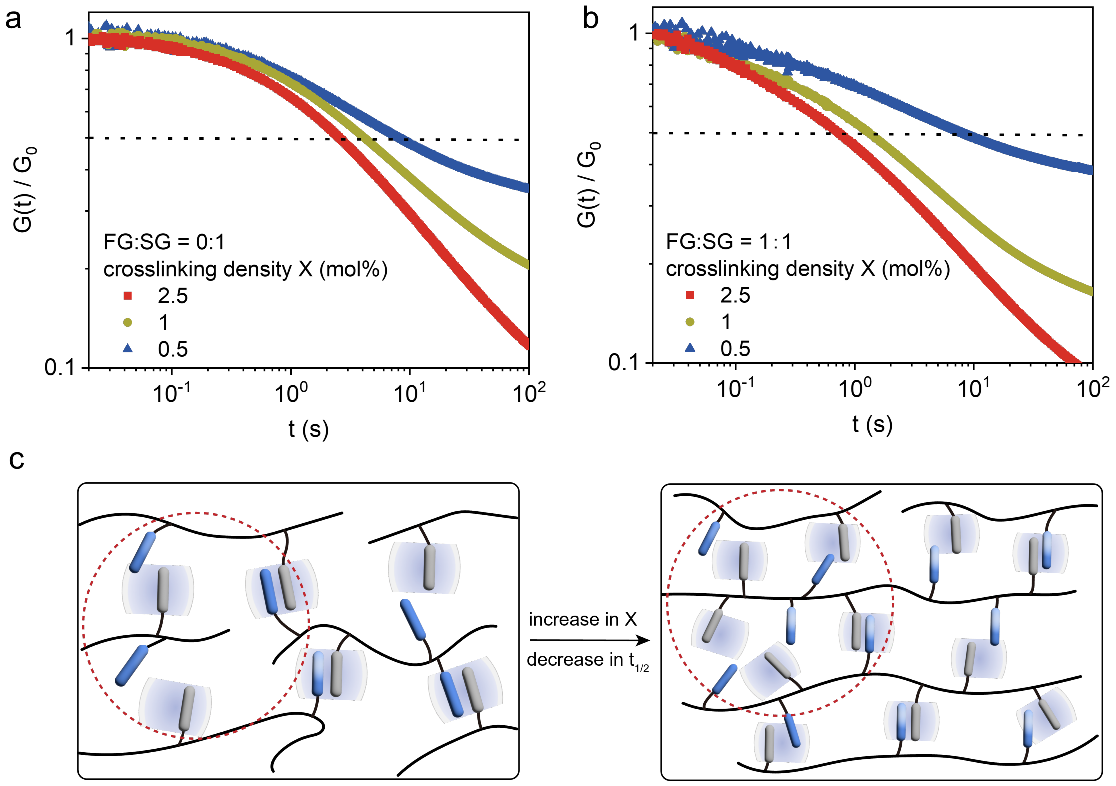

Supplementary Figure 6: Effect of host–guest crosslinking density on the stress relaxation behavior of SPNs. **(a)** Stress relaxation profiles of SPN 1 at crosslinking density X = 0.5, 1, and 2.5 mol%. **(b)** Stress relaxation profiles of SPN 3 at crosslinking density X = 0.5, 1, and 2.5 mol%. **(c)** Schematic illustration of the effect of host–guest non-covalent crosslinking density X on the stress relaxation behavior of SPNs. The monomer concentration C_M = 2 M.

Increasing crosslink density accelerates stress relaxation primarily through architectural effects rather than changes in individual bond dissociation kinetics. At fixed supramolecular exchange rates, a higher density of dynamic crosslinks reduces the polymer strand length between crosslinks (smaller N). According to Rouse-type dynamics ($\tau_{\text{Rouse}} \sim N^2$), shorter strands possess intrinsically faster relaxation times, enabling more rapid stress relaxation through local chain rearrangements. Additionally, shorter network strands and reduced topological length scales facilitate more efficient reconfiguration of transient constraints, further contributing to accelerated macroscopic stress relaxation.

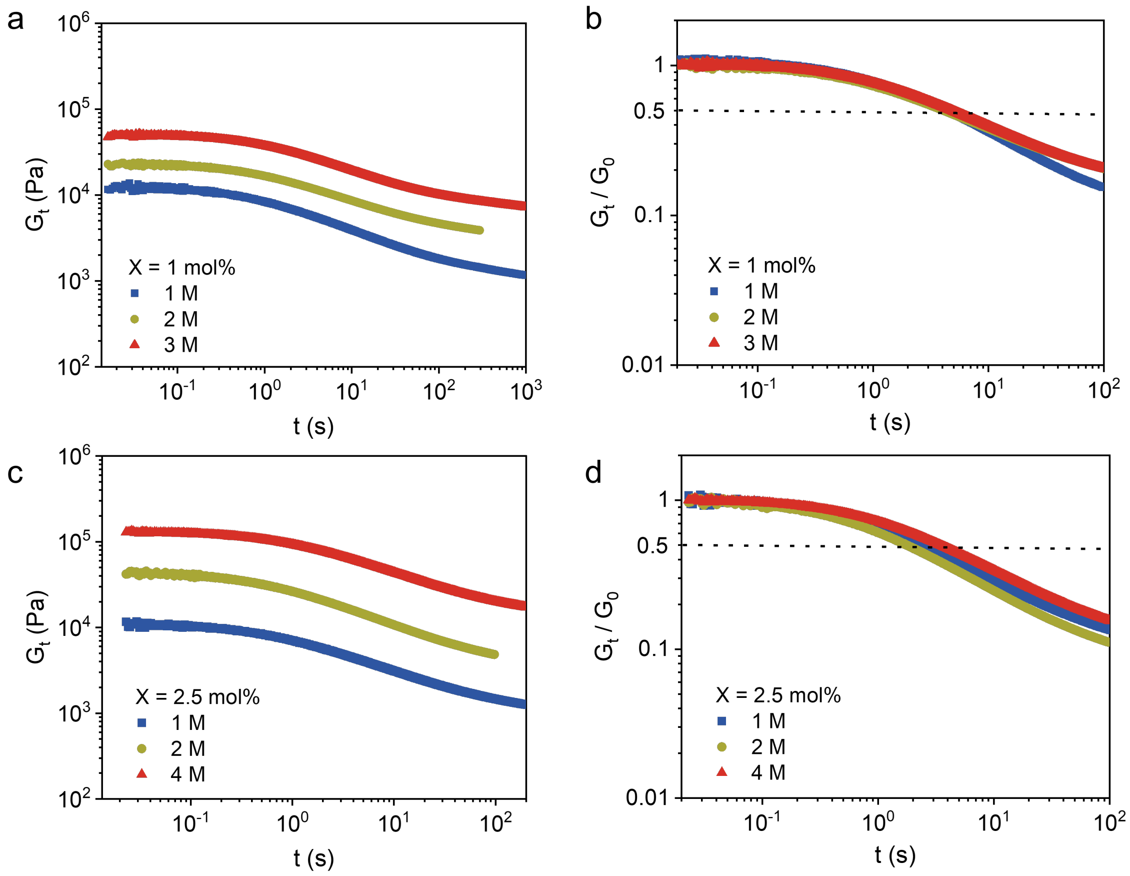

Supplementary Figure 7: Effect of monomer concentration on the stress relaxation behavior of SPNs. **(a)** Stress relaxation profiles of SPN 1 with monomer concentration C_M = 1 M, 2 M, and 4 M at a crosslinking density X = 1 mol%. **(b)** Corresponding normalized stress relaxation curves for (a). **(c)** Stress relaxation profiles of SPN 1 with monomer concentration C_M = 1 M, 2 M, and 3 M at a crosslinking density X = 2.5 mol%. **(d)** Corresponding normalized stress relaxation curves for (c).

Figure S7a shows that the stress relaxation moduli $G(t)$ of SPN 1 increase as the monomer concentration rises from 1 M to 3 M at a fixed crosslinking density X = 1 mol%. However, the curves remain parallel to each other. When normalized by the initial modulus G_0, the stress relaxation curves overlap (Figure S7b), indicating an identical relaxation rate. A similar trend is observed for SPNs at a crosslinking density X = 2.5 mol%, further confirming that stress relaxation is independent of monomer concentration. This suggests that host–guest interactions primarily govern the relaxation dynamics rather than polymer chain concentration. At a fixed crosslinking density, the density of non-covalent crosslinks remains constant, meaning that crosslinks are likely to exchange with surrounding partners at the same probability, leading to a uniform stress relaxation rate. This independence of stress relaxation dynamics from polymer concentration has also been observed in other dynamic hydrogels.[3]

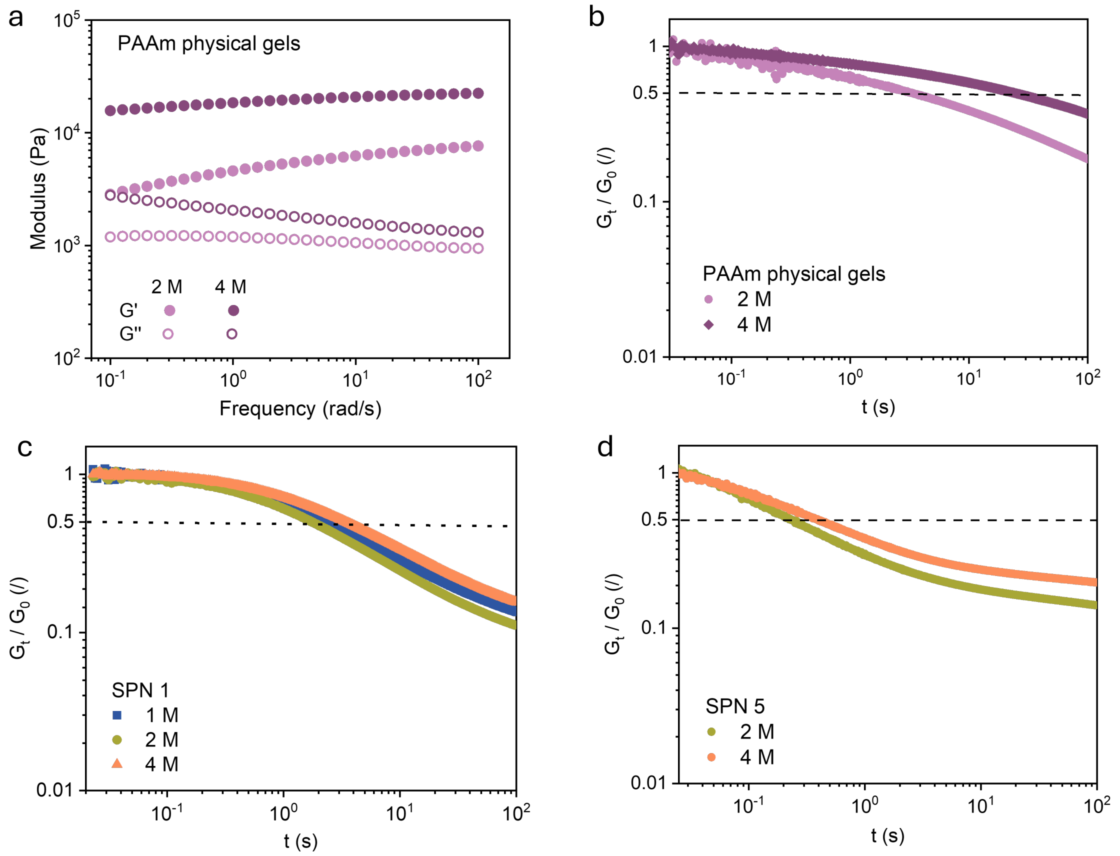

Supplementary Figure S8: Effect of entanglement on stress-relaxation behavior of PAAm networks. **(a)** Frequency sweeps of PAAm physical gels (no chemical crosslinks) at monomer concentrations C_M = 2 M and 4 M, showing more solid-like behavior and slower dynamics at higher concentration. **(b)** Normalized stress-relaxation curves of PAAm physical gels at C_M = 2 M and 4 M, demonstrating slower relaxation at higher concentration due to increased chain constraints and entanglement. **(c)** Normalized stress-relaxation curves of SPN 1 at monomer concentrations C_M = 1–4 M. **(d)** Normalized stress-relaxation curves of SPN 5 at monomer concentrations C_M = 2 M and 4 M.

To assess the influence of entanglement on stress relaxation, we measured polymerized PAAm physical gels with different monomer concentrations (C_M = 2 M and 4 M) in the absence of supramolecular crosslinks (Figure S8a-b). Both samples exhibit gel-like behavior with $G' > G''$ over the measured frequency range. Increasing monomer concentration leads to higher modulus and slower stress relaxation, with the stress-relaxation half-time increasing from approximately 3 s at 2 M to 30 s at 4 M, consistent with reduced polymer chain mobility at higher entanglement density. In contrast, in supramolecular polymer networks (SPNs) stress relaxation is dominated by the dissociation–association kinetics of the host–guest crosslinks rather than by polymer chain relaxation. SPN 1 (slower exchange kinetics) exhibits a characteristic relaxation half-time of approximately 3 s that is largely independent of monomer concentration (Figure S8c), while SPN 5

relaxes an order of magnitude faster ($t_{1/2} \approx 0.3$ s) due to its faster supramolecular exchange kinetics, again with minimal dependence on monomer concentration (Figure S8d). Although the physical PAAm gel at $C_{\mathrm{M}} = 2$ M and SPN 1 exhibit similar relaxation half-times, the corresponding moduli differ by several orders of magnitude. The physical gel relaxes stress at a modulus of $\sim 10^3$ Pa, whereas SPN 1 relaxes stress at a modulus of $\sim 10^5$ Pa, highlighting the decoupling of elastic stiffness and relaxation timescale achieved through supramolecular kinetic control. Together, these results show that while increased entanglement density strongly slows relaxation in chain-controlled physical gels, the stress-relaxation timescale in SPNs is primarily dictated by supramolecular crosslink kinetics, with polymer concentration mainly affecting modulus and toughness.

Influence of Network Quality on Toughness and Relaxation

In polymer networks, network quality, such as the presence of dangling or elastically inactive chains, can influence elastic efficiency and contribute to secondary dissipative processes (e.g., chain pull-out or frictional interactions). In the SPNs presented here, however, the impact of such network imperfections on toughness is secondary to the effects of supramolecular crosslink lifetime and polymer concentration, which govern the magnitude and rate of energy dissipation during fracture. Importantly, the stress-relaxation behavior in SPNs is dictated primarily by host-guest dissociation-association kinetics rather than by network topology or polymer chain mobility. Variations in monomer concentration, which may alter network connectivity and the fraction of elastically inactive chains, have minimal impact on the relaxation half-time $t_{1/2}$, whereas modifications of supramolecular exchange kinetics shift the relaxation timescale by nearly an order of magnitude.

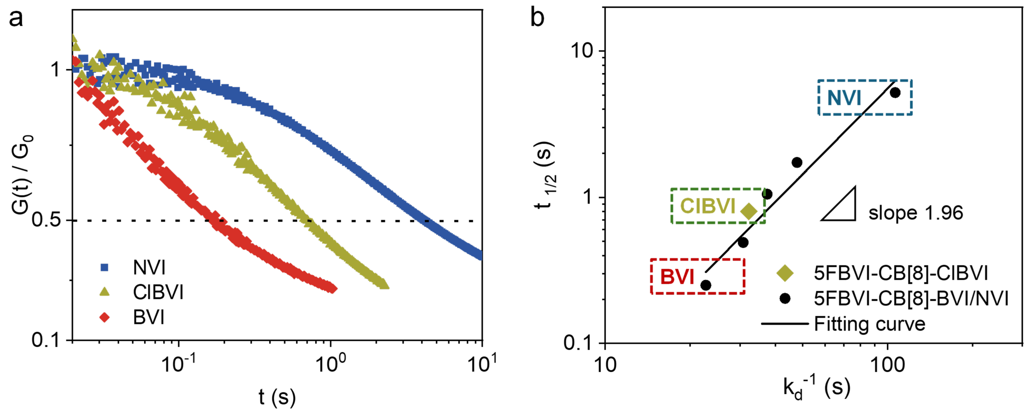

Supplementary Figure 9: Stress relaxation behavior of SPNs incorporating a second guest, 1-(4-chlorobenzyl)-3-vinylimidazolium bromide (ClBVI), compared to fast guest–BVI and slow guest–NVI. (a) Normalized stress relaxation curves. **(b)** Scaling relationship between stress relaxation dynamics and bond lifetime:$t_{1/2}$ as a function of k_d^{-1}. The monomer concentration C_M = 2 M, and the crosslinking density X = 1 mol%.

Figure S9a shows that SPN 1exhibits the slowest stress relaxation behavior, while SPN 5 exhibits the fastest, with ClBVI-SPN falling between the two, demonstrating an intermediate stress relaxation rate. Figure S9b presents $t_{1/2}$ as a function of the bond lifetime, where ClBVI-SPN aligns with the scaling relationship of $t_{1/2}$ to k_d^{-1} (with a slope of 1.96) observed in SPN 1–5 systems. Given that ClBVI has a dissociation rate constant of $k_d \approx 0.031$ s^{-1} ($k_d^{-1} \approx 32$ s), corresponding to a 3:2 ratio of fast:slow guest.

Origin of the Quadratic Scaling between τ and $t_{1/2}$:

Across formulations at fixed chemical compositions, we observe a near-quadratic scaling, $t_{1/2} \propto \tau^2$, where $\tau = k_d^{-1}$ is the bond lifetime. This super-linear dependency is consistent with a relaxation pathway that requires correlated, multi-event dissociation-reassociation and strand/network reconfiguration, rather than being governed by a single bond dissociation event. In this sense, macroscopic stress relaxation reflects cooperative, network-level rearrangements that lead to a characteristic relaxation timescale growing faster than linearly with τ. We emphasize that the observed exponent is phenomenological rather than universal, reflecting effective network-level kinetic amplification rather than a specific microscopic rate law. This framework differs from conventional associative polymer models, in which macroscopic relaxation is typically assumed to scale approximately linearly with the lifetime of an individual sticker ($t_{relax} \sim \tau$).[4] In CB[8] SPNs, the relatively high equilibrium binding constant of the second guest complexation ($K_{eq} \approx 10^4$-10^5 M^{-1}) can promote frequent rebinding, making cooperative network-level rearrangements more influential than isolated bond breaking in setting the macroscopic relaxation time.

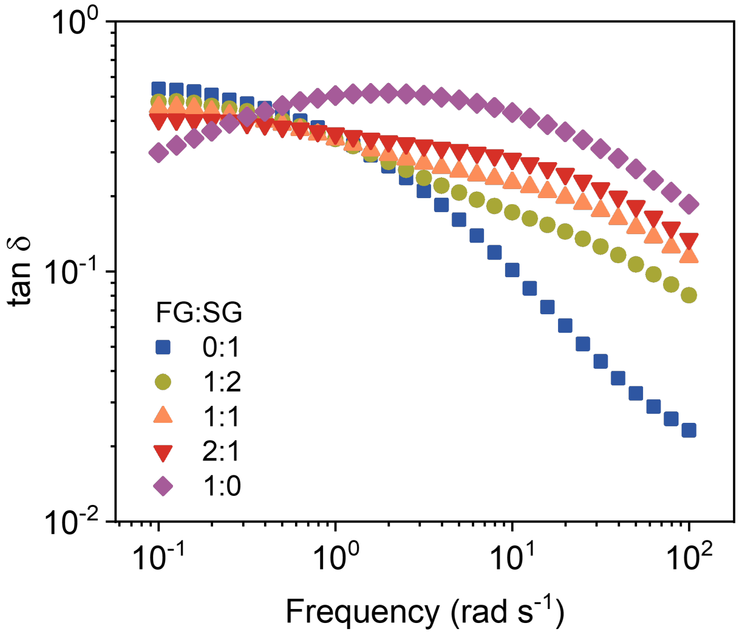

Supplementary Figure 10: Viscoelastic behavior showing $\tan\delta$ **(G''/G') as a function of frequency for SPNs.** The monomer concentration $C_{\mathrm{M}} = 2$ M and the crosslinking density $X = 1$ mol%.

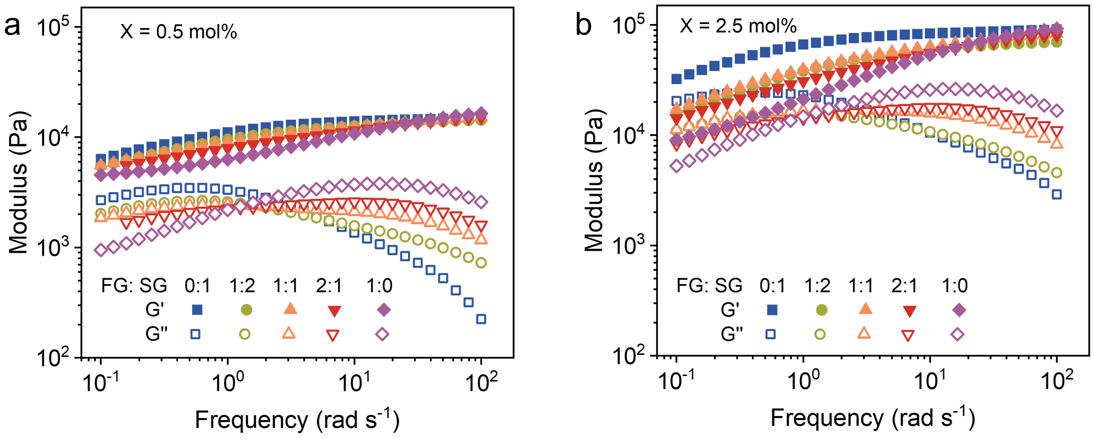

Supplementary Figure 11: Frequency sweep measurements for SPNs with varying fast:slow guest ratios. **(a)** crosslinking density $X = 0.5$ mol% and **(b)** $X = 2.5$ mol%.

Figure S11 shows the frequency sweep measurements of SPNs with varying fast:slow guest ratios. As the proportion of BVI increases, the SPNs transition from glass-like to rubber-like viscoelastic behavior. Additionally, increasing the crosslinking density from 0.5 mol% to 2.5 mol% results in elevated values of both G' and G'', indicating that higher crosslinking densities enhance the modulus and lead to mechanically stronger materials.

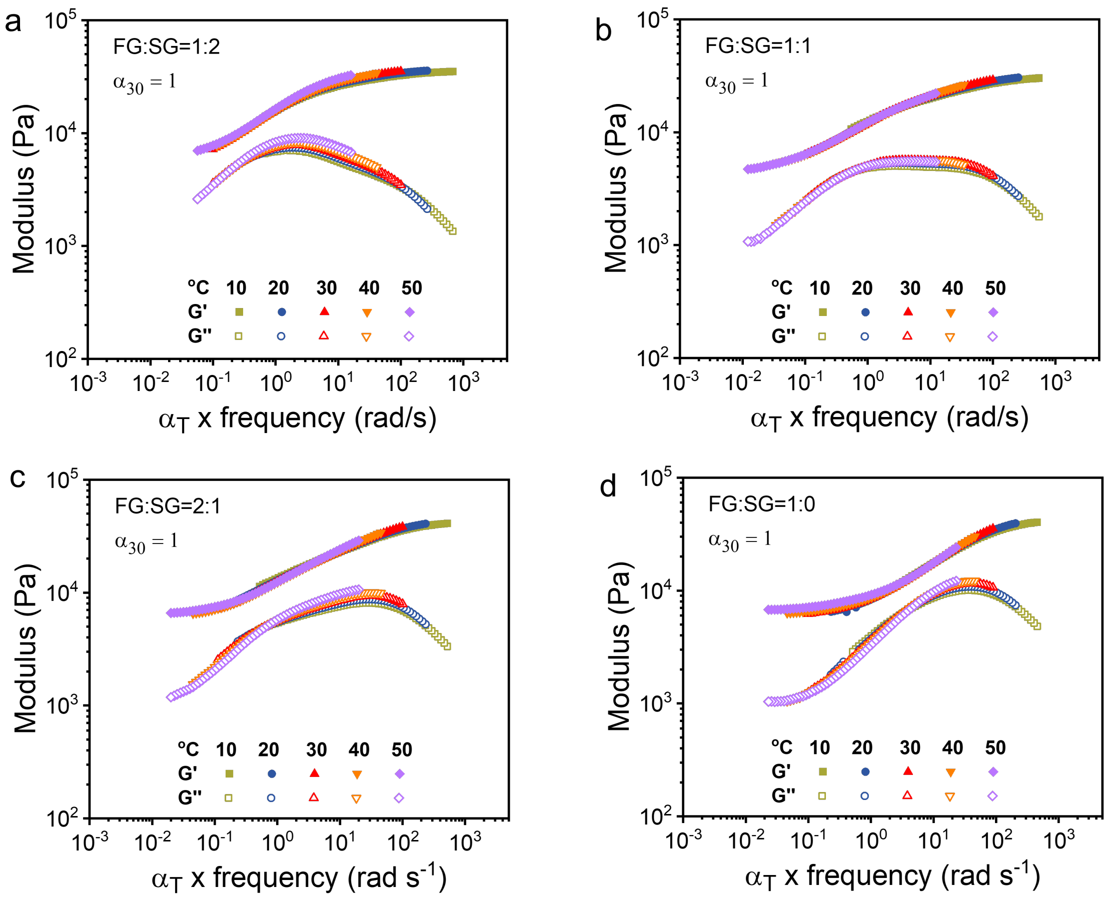

Supplementary Figure 12: Master curves from time–temperature superposition (TTS) for SPNs. (a) SPN 2. **(b)** SPN 3. **(c)** SPN 4. **(d)** SPN 5. The monomer concentration $C_{\mathrm{M}} = 2$ M and the crosslinking density $X = 1$ mol%.

Figure S12 presents the master curves of storage modulus (G') and loss modulus (G'') obtained via time–temperature superposition (TTS) for SPNs with varying fast:slow guest ratios, referenced at 30 °C (with shift factor $\alpha_T = 1$). As the fast:slow guest ratio increases, the viscoelastic behavior of the SPNs transitions from glass-like to rubber-like. This is evidenced by the shift of the G'' peak from low to high frequency, attributed to a change in the dissociation kinetics of the host–guest crosslinks from slow to fast.

The temperature dependence of the shift factor α_T was used to extract the activation energy (E_a) using the Arrhenius equation:

$$\alpha_T = Ae^{E_a/RT}$$

where α_T is the horizontal shift factor, R is the ideal gas constant (8.314 J·mol^{-1}·K^{-1}), T is temperature in Kelvin, and A is a pre-exponential factor. The calculated E_a values for SPNs with BVI:NVI ratios of 1:0, 2:1, 1:1, 1:2, and 0:1 range from 53 to 67 kJ/mol (13–16 kcal/mol).

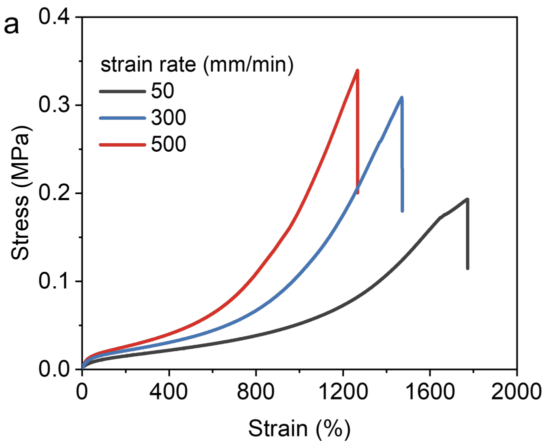

Supplementary Figure 13: Uniaxial stress–strain curves of SPNs under different strain rates (50, 300, and 500 mm/min) (a) SPN 5 (1:0 BVI:NVI) and **(b)** SPN 1 (0:1 BVI:NVI). The crosslinking density $X = 1$ mol% and the monomer concentration $C_{\mathrm{M}} = 2$ M.

Figure S13 shows the tensile behavior of the SPNs under different applied strain rates. Both the Young's modulus and fracture strength increase with increasing strain rate. This strong strain-rate dependence is consistent with the previously observed correlation between frequency and moduli, and both can be attributed to the dynamic nature of the reversible crosslinking motifs.

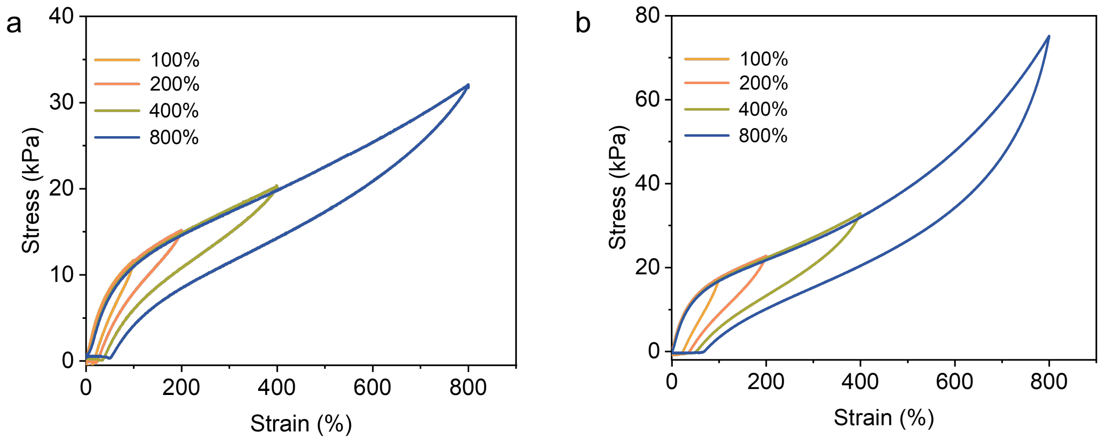

Supplementary Figure 14: Loading–unloading curves and hysteresis behavior of SPNs **(a)** SPN 5 and **(b)** SPN 2 at maximum strains of 100%, 200%, 400%, and 800%. The crosslinking density X = 1 mol% and the monomer concentration C_M = 2 M.

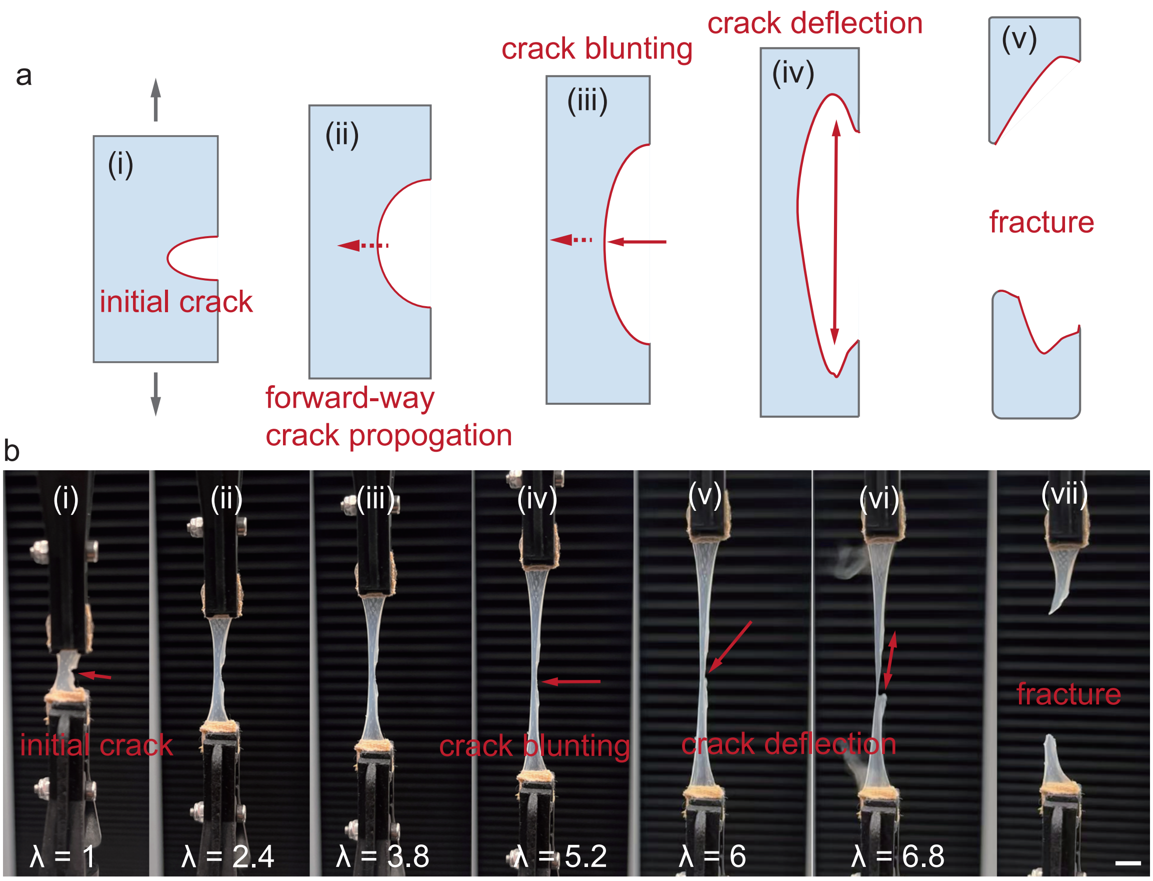

Supplementary Figure 15. Fracture behavior of pre-cut SPNs under uniaxial stretching. (a) Schematic illustration of pre-cut SPN 1 subjected to uniaxial stretch until fracture. (i) Initial state with a pre-existing notch. (ii) Expected forward crack propagation indicated by the red dashed arrow. (iii) Crack blunting. (iv) Crack deflection, where the crack deviates and propagates sideways along the stretch direction. (v) Final fracture. The black arrows indicate stretch direction. (b) Digital images of notched SPN 1 under uniaxial stretching, C_M = 2 M, X = 2.5 mol%. Crack blunting occurs at large deformation (iv). Crack deflection begins in (v), where a secondary crack propagates along the stretch direction, deviating from the initial crack path and leading to a large crack (vi). The scale bar represents 5 mm.

Figure S15 illustrates the fracture behavior of pre-cut SPNs under uniaxial stretching. An initial notch approximately 1 mm in size was introduced to each specimen. Upon deformation, the crack tip begins to blunt at high stretch ratios, as shown in Figure S15a(iii) and b(iv) at λ = 5.2. Following this blunting, crack deflection occurs: a secondary crack propagates along the direction of stretching, deviating from the original crack path and ultimately forming a larger crack. Eventually, the SPN specimen fractures, exhibiting rough fracture surfaces. This phenomenon of crack deflection has also been observed in mechanically robust biological tissues, elastomers, and hydrogels, where it serves as an effective toughening mechanism.[5–8]

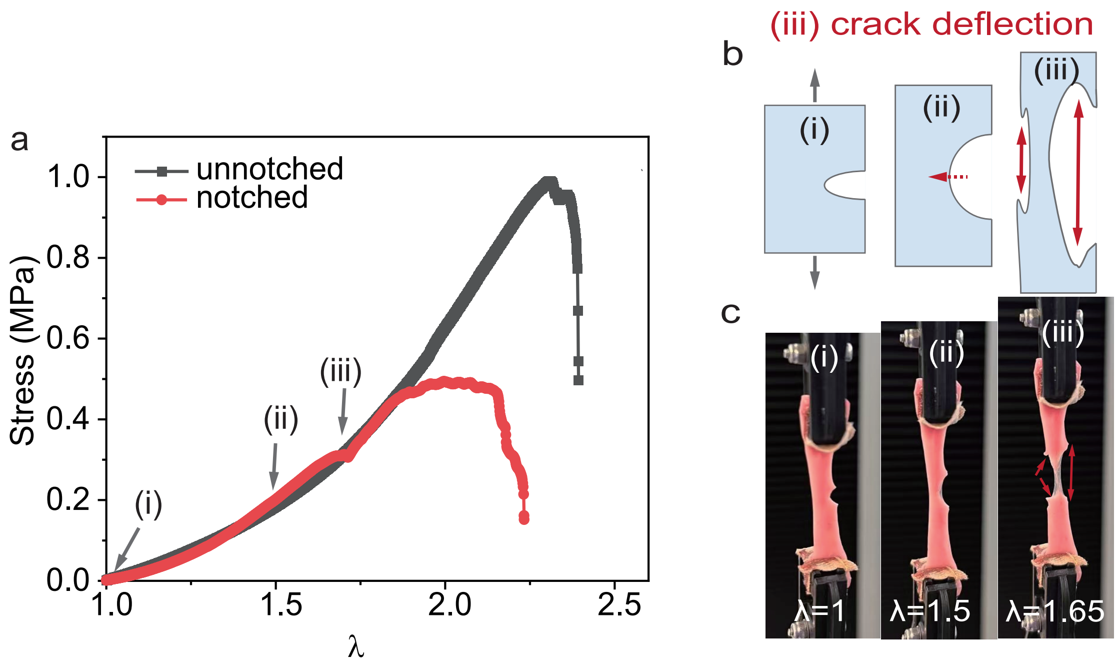

Supplementary Figure 16. Fracture behavior of pre-cut pig heart vessel under uniaxial stretching. (a) Schematic illustration of a notched pig heart vessel being stretched until fracture. (i) Initial state with a pre-existing notch. (ii) Expected forward crack propagation direction indicated by the red dashed arrow. (iii) Observed crack deflection, where the crack propagates sideways along the stretch direction. (b) Stress–strain curves for notched and unnotched pig heart vessels. (c) Digital images of the notched pig heart vessel under uniaxial stretching, showing progression toward fracture.

Figure S16 presents the fracture behavior of pre-cut pig heart vessels under uniaxial stretching. When the specimen is stretched to a moderate strain level ($\lambda = 1.65$), crack deflection occurs: the crack propagates parallel to the stretch direction (Figure S16a(iii) and b(iii)), rather than continuing forward along the initial notch direction (indicated by the red dashed arrow in Figure S16a(ii)). The fracture energy of the pig heart vessel is calculated to be approximately 1600 J/m^2.

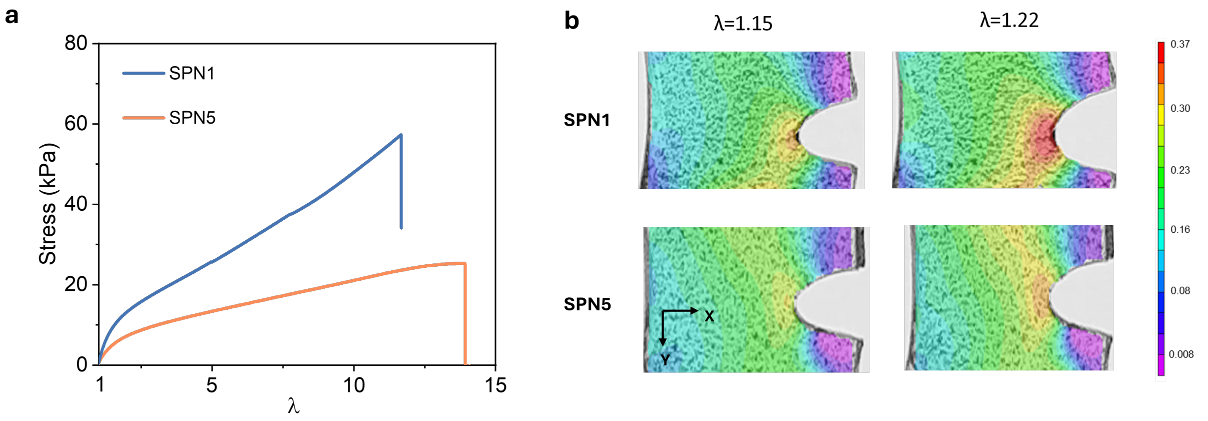

Supplementary Figure 17: Tensile fracture behavior and DIC analysis of SPNs. **(a)** Tensile stress–stretch curves for SPN 1 and SPN 5 containing a pre-existing crack, tested at a stretch rate of 25 mm min^{-1}. **(b)** Representative Digital Image Correlation (DIC) strain maps (ε_{yy}) of SPN 1 and SPN 5 at identical global stretches ($\lambda = 1.15$ and $\lambda = 1.22$), illustrating more localized crack-tip strain concentration in SPN 1 compared to a broader deformation field in SPN 5.

To provide spatially resolved experimental evidence of strain distribution, Digital Image Correlation (DIC) measurements were performed on SPN 1 and SPN 5. A random speckle pattern was applied to the hydrogel surface using matte black acrylic paint following established procedures,[9] and samples were imaged during tensile deformation using a fixed camera setup. The corresponding stress–stretch curves and representative DIC strain maps are shown in Figure S17. At comparable global stretches ($\lambda = 1.15$ and $\lambda = 1.22$), SPN 1 sustains a higher tensile stress and exhibits a more localized crack-tip strain concentration than SPN 5, whereas SPN 5 displays a broader and more diffuse deformation field near the crack tip. These DIC results qualitatively demonstrate that slower supramolecular dissociation kinetics give rise to more confined crack-tip deformation and more intense local dissipation, consistent with the trends inferred from bulk hysteresis and fracture measurements.

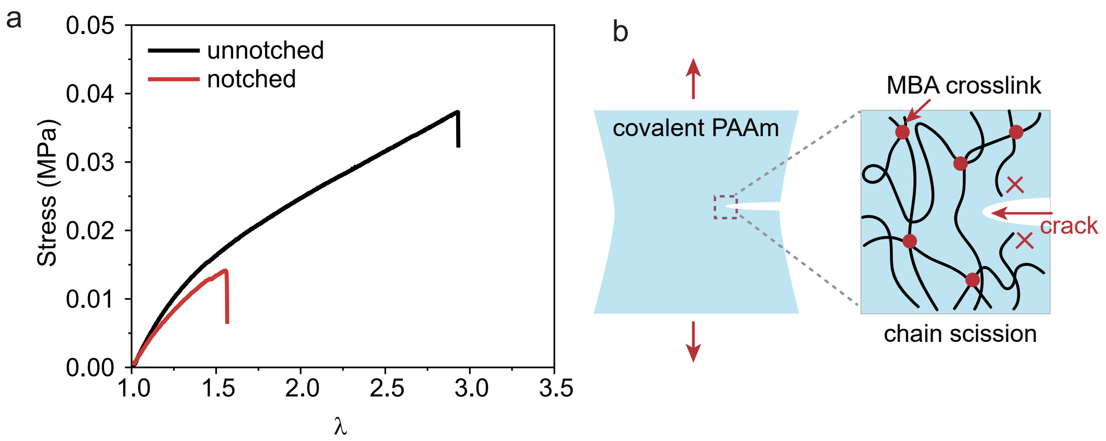

Supplementary Figure 18: Fracture behavior of MBA covalently crosslinked PAAm hydrogels (a) Uniaxial stress–strain curves for unnotched and notched samples. The monomer concentration $C_{\mathrm{M}} = 2$ M and the crosslinking density X (MBA) = 0.5 mol%. **(b)** Schematic of fracture behavior of a covalently crosslinked PAAm hydrogel with a crack. The fracture energy G_c was calculated to be 26 J/m^2.

Figure S18a presents the stress–strain curve of MBA-crosslinked PAAm hydrogels for both notched and unnotched samples. The fracture strength (σ_c) of the unnotched sample is around 0.04 MPa (40 kPa) with a critical stretch ratio λ_c of 3, while the notched sample fractures at a much lower strength (0.015 MPa) and reduced λ_c of 1.6. The fracture energy G_c was calculated to be 26 J/m^2. Compared to SPNs at the same monomer concentration ($C_{\mathrm{M}} = 2$ M) and crosslinking density ($X = 0.5$ mol%) as shown in Table S1, these results indicate that covalent PAAm hydrogels are mechanically weak due to the lack of effective energy dissipation within the network, such that chain scission occurs easily at the crack front, as illustrated in Figure S18b.

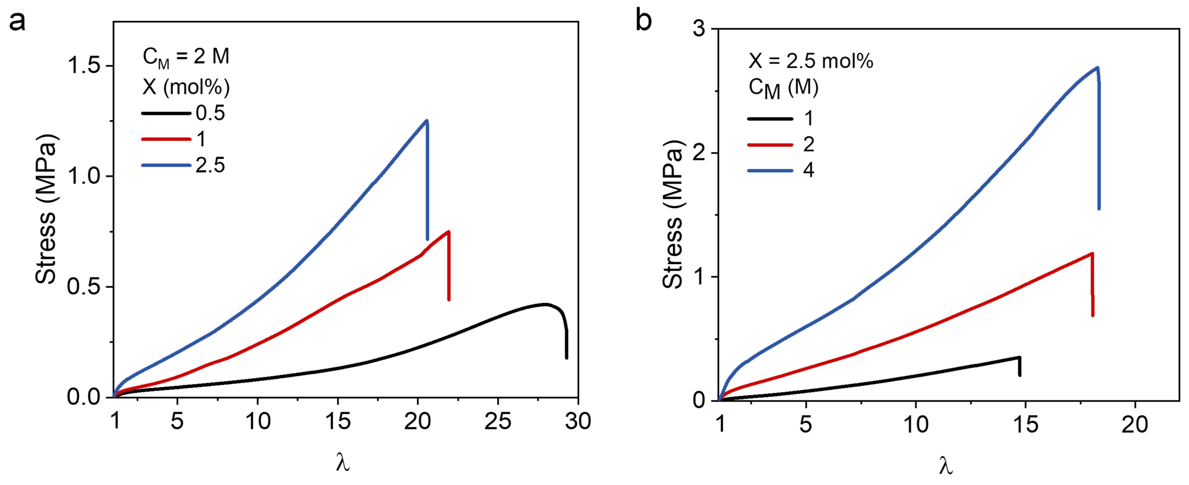

Supplementary Figure 19: The effect of crosslinking densities and monomer concentrations to mechnical properties of SPNs (a) Uniaxial stress–strain curves of SPN 1 with various crosslinking densities (X = 0.5, 1, and 2.5 mol%) at a fixed monomer concentration C_{M} = 2 M. **(b)** Uniaxial stress–strain curves of SPNs1 with various monomer concentrations (C_{M} = 1, 2, and 4 M) at a fixed crosslinking density X = 2.5 mol%.

Figure S19a shows the uniaxial tensile stress–stretch behavior of SPN 1 with varying crosslinking densities (X = 0.5, 1.0, and 2.5 mol%) at a fixed monomer concentration (C_{M} = 2 M). As the crosslinking density increases, both the Young's modulus and fracture stress increase significantly. The SPN with the highest crosslink density (X = 2.5 mol%) exhibits the highest fracture stress of 1.25 MPa, reflecting enhanced network stiffness and strength. In contrast, the sample with X = 0.5 mol% shows a much lower stress response but a higher stretch ratio (λ_c = 30), indicating greater extensibility. These results confirm that increasing the density of dynamic crosslinks enhances the mechanical robustness of the SPNs.

Figure S19b shows that increasing the monomer concentration (C_{M}) also leads to significant increases in both the Young's modulus and the maximum stress at failure. The SPN with C_{M} = 4 M exhibits the highest fracture stress of 2.68 MPa, indicating a denser polymer network with greater load-bearing capacity. These results demonstrate that increasing the monomer concentration reinforces the network by promoting additional chain entanglements and crosslink formation, thereby enhancing the mechanical strength of the SPNs.

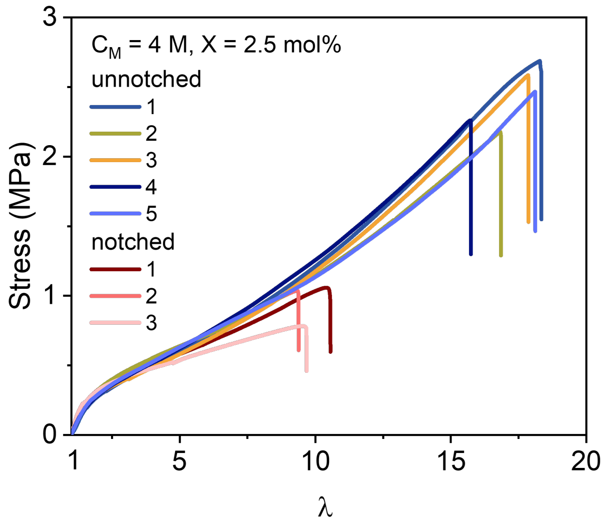

Supplementary Figure 20: Uniaxial stress–strain curves of SPN 1 for unnotched and notched samples. The monomer concentration C_{M} = 4 M and the crosslinking density X = 2.5 mol%. At least three replicate samples were tested.

Figure S20 presents the uniaxial stress–strain curves for notched and unnotched SPN 1 at C_{M} = 4 M and X = 2.5 mol%. The fracture toughness (G_c) of the single-edge notched SPNs was calculated according to the standard method for soft polymer networks,[10]

$$G_c = \frac{6WC_0}{\sqrt{\lambda_c}},$$

where λ_c is the critical fracture stretch ratio at which the edge-notched sample ruptures under uniaxial tension, W is the strain energy density of an uncracked sample stretched to λ_c, and C_0 is the initial crack length. At least three replicate samples were tested to ensure reproducibility. The resulting fracture energy, G_c = 14,500 J m^{-2}, represents an exceptionally high value among supramolecular hydrogels and is attributed to the slow-dissociating crosslink dynamics combined with polymer-chain entanglement and network cooperativity.

Table S1: Benchmark mechanical and relaxation properties of representative covalent, hybrid, and supramolecular hydrogels used for comparison in Fig. 1d.

**Material system**	**Composition / crosslink type**	$t_{1/2}$ **(s)**	G_c **(J m^{-2})**	**Modulus (kPa)**	**Notes**
Covalent PAAm gel[10]	Covalent	> 1000	~ 10	10–20	Weak, slow relaxation
DN hydrogel[11]	Covalent	> 1000	10^3–10^4	100–300	Tough, slow
PAAm–alginate[12]	Covalent and non-covalent	> 100	10^3–10^4	50–200	Tough, moderate relaxation
DNA hydrogel[13]	DNA (non-covalent)	< 10	$< 10^2$	0.1–1	Weak, fast relaxation
Blood vessel	Native tissue	3	1500	17.5	Strong, moderately viscoelastic
Muscle	Native tissue	10	1000	20	Strong, compliant
**SPN (this work)**	Host–guest	0.1–100	10^3–10^4	20–140	Tough and fast

Ranges are approximate and depend on composition, crosslink density, water content, and testing protocols. Values are provided for contextual comparison rather than direct one-to-one benchmarking. Across reported systems, mechanically robust covalent or multi-network hydrogels typically exhibit slow stress relaxation, whereas rapidly relaxing supramolecular systems often display limited fracture resistance. The present SPNs are positioned within this landscape to illustrate the simultaneous combination of rapid relaxation and high fracture toughness within a single supramolecular network platform.

Table S2: Summary of water content and mechanical properties of supramolecular polymer networks (SPNs) in this work, compared with representative biological tissues and classical tough hydrogel architectures reported in the literature.

**Reference**	**Components**	C_M **(M)**	**Water content (wt%)**	σ_c **(MPa)**	G_c **(J m^{-2})**
DN Gel[11]	PAMPS–PAAm	4	83	0.35	1000
Ionically-crosslinked Gel[14]	P(NaSS–MPTC)	2	50	1.8	4000
Entanglement Gel[15]	PAAm	3	70	0.39	2000
SR Gel[16]	PEG–CD	–	70	0.31	2200
Heart muscle	–	–	75–80	0.5	1000
Blood vessel	–	–	75–80	0.7	1500
This work (SPN-a)	SPN	2	82.6	0.83	3340
This work (SPN-b)	SPN	2	79.1	1.25	6570
This work (SPN-c)	SPN	4	60.4	2.68	14500

C_M denotes the total monomer concentration used during gel preparation, as reported in the original references. DN, double network; PAAm, polyacrylamide; NaSS, sodium p-styrenesulphonate; MPTC, 3-(methacryloylamino)propyl-trimethylammonium chloride; SR, slide-ring; PEG, polyethylene glycol; CD, hydroxypropyl-α-cyclodextrin; SPN, supramolecular polymer networks in this work. SPN-a, SPN-b, and SPN-c correspond to crosslinking densities X = 1, 2.5, and 2.5 mol%, respectively, using the 5FBVI–CB[8]–NVI host–guest system (SPN-c prepared at higher C_M).

Table S2 summarizes the water content and mechanical properties of SPNs in comparison with representative classical tough hydrogels and biological tissues. Increasing the monomer concentration from 2 to 4 M reduces the water content from 82.6 wt% to 60.4 wt%, corresponding to a higher polymer volume fraction and a denser load-bearing network. This reduction in hydration is accompanied by a marked increase in fracture energy, consistent with structural reinforcement by higher polymer content. Even at a moderate monomer concentration of 2 M (water content ~80 wt%), SPNs exhibit fracture energies of 3340–6570 J m^{-2}, comparable to or exceeding many classical tough hydrogel systems prepared at similar solid contents. Across this hydration range, the stress-relaxation half-time remains largely invariant, indicating that viscoelastic timescales are governed predominantly by host–guest dissociation kinetics rather than solvent content.

Table S3: Supplementary Table S3: Kinetic programming landscape across dynamic crosslink chemistries. Representative association/dissociation constants and relaxation half-times for common dynamic chemistries, alongside the CB[8] host–guest system used in this work.

**Dynamic chemistry / interaction**	k_a (M^{-1} s^{-1})	k_d (s^{-1})	K (M^{-1})	$t_{1/2}$ (s)
Imine	10^1–10^3	10^{-1}–10^1	10^1–10^4	< 10
Hydrazone (hydrazine)	10^{-2}–10^1	10^{-6}–10^{-4}	10^6–10^8	> 1000
Oxime	10^{-1}	< 10^{-9}	> 10^6	> 10^4
Boronate ester	7–15	10^{-2}–10^{-1}	10^2–10^2	< 10
Thioester	1.2–1.7	—	—	> 10^4
β-Cyclodextrin/adamantane	10^8	10^3	10^5	< 30
Alginate–Ca^{2+} coordination	—	—	10^4	50–10^4
**CB[8] host–guest (this work)**	10^3	10^{-3}–10^{-2}	10^4–10^5	0.1–100

Ranges are representative values compiled from literature (see Table 3 and refs.[17]) and may vary with pH, ionic strength, temperature, and substituents.
Values for **CB[8]** correspond to this work (main text and SI). Definition: $K = k_a/k_d$.

Table S3 compiles representative association/dissociation rates (k_a, k_d) and equilibrium constants ($K = k_a/k_d$) for common dynamic crosslinks. Dynamic covalent motifs typically have small k_d (long lifetimes; slow relaxation), whereas fast host–guest pairs (e.g., β-CD/adamantane) have large k_d (short lifetimes; rapid relaxation). The CB[8] complex lies between these limits, balancing stability and exchange. In transient networks, k_d sets the microscopic breaking rate of load-bearing crosslinks and thus the intrinsic relaxation clock, while K and formulation (stoichiometry, concentration) determine the bound fraction and rebinding probability.

References

[1] Huang, Z. *et al.* Host-enhanced phenyl-perfluorophenyl polar- π interactions. *J. Am. Chem. Soc* **142**, 7356–7361 (2020).

[2] Huang, Z. *et al.* Highly compressible glass-like supramolecular polymer networks. *Nat. Mater.* **21**, 103–109 (2022).

[3] Lou, J., Friedowitz, S., Will, K., Qin, J. & Xia, Y. Predictably engineering the viscoelastic behavior of dynamic hydrogels via correlation with molecular parameters. *Adv. Mater.* **33**, 2104460 (2021).

[4] Rubinstein, M. & Semenov, A. N. Dynamics of entangled solutions of associating polymers. *Macromolecules* **34**, 1058–1068 (2001).

[5] Luo, F. *et al.* Crack blunting and advancing behaviors of tough and self-healing polyampholyte hydrogel. *Macromolecules* **47**, 6037–6046 (2014).

[6] Sun, J.-Y. *et al.* Highly stretchable and tough hydrogels. *Nature* **489**, 133–136 (2012).

[7] Wei, Y., Ju, J., Creton, C. & Narita, T. Unexpected fracture behavior of ultrasoft associative hydrogels due to strain-induced crystallization. *ACS Macro Lett.* **12**, 1106–1111 (2023).

[8] Lee, S. & Pharr, M. Sideways and stable crack propagation in a silicone elastomer. *Proc. Natl. Acad. Sci. U.S.A.* **116**, 9251–9256 (2019).

[9] Liu, M., Guo, J., Hui, C.-Y. & Zehnder, A. Application of digital image correlation (dic) to the measurement of strain concentration of a pva dual-crosslink hydrogel under large deformation. *Exp. Mech.* **59**, 1021–1032 (2019).

[10] Long, R. & Hui, C.-Y. Fracture toughness of hydrogels: measurement and interpretation. *Soft Matter* **12**, 8069–8086 (2016).

[11] Gong, J. P., Katsuyama, Y., Kurokawa, T. & Osada, Y. Double-network hydrogels with extremely high mechanical strength. *Adv. Mater.* **15**, 1155–1158 (2003).

[12] Sun, J.-Y. *et al.* Highly stretchable and tough hydrogels. *Nature* **489**, 133–136 (2012).

[13] Peng, Y.-H. *et al.* Dynamic matrices with dna-encoded viscoelasticity for cell and organoid culture. *Nat. Nanotechnol.* **18**, 1463–1473 (2023).

[14] Sun, T. L. *et al.* Physical hydrogels composed of polyampholytes demonstrate high toughness and viscoelasticity. *Nat. Mater.* **12**, 932–937 (2013).

[15] Kim, J., Zhang, G., Shi, M. & Suo, Z. Fracture, fatigue, and friction of polymers in which entanglements greatly outnumber cross-links. *Science* **374**, 212–216 (2021).

[16] Liu, C. *et al.* Tough hydrogels with rapid self-reinforcement. *Science* **372**, 1078–1081 (2021).

[17] Lou, J. & Mooney, D. J. Chemical strategies to engineer hydrogels for cell culture. *Nat. Rev. Chem.* **6**, 726–744 (2022).
